# Resistance gene expression determines the in vitro chemosensitivity of non-small cell lung cancer (NSCLC)

**DOI:** 10.1186/1471-2407-9-300

**Published:** 2009-08-27

**Authors:** Sharon Glaysher, Dennis Yiannakis, Francis G Gabriel, Penny Johnson, Marta E Polak, Louise A Knight, Zoe Goldthorpe, Katharine Peregrin, Mya Gyi, Paul Modi, Joe Rahamim, Mark E Smith, Khalid Amer, Bruce Addis, Matthew Poole, Ajit Narayanan, Tim J Gulliford, Peter E Andreotti, Ian A Cree

**Affiliations:** 1 Translational Oncology Research Centre, Queen Alexandra Hospital, Portsmouth PO6 3LY, UK; 2 Departments of Thoracic Surgery, Oncology and Pathology, Derriford Hospital, Plymouth, UK; 3 Departments of Thoracic Surgery and Pathology, Southampton General Hospital, Southampton, UK; 4 School of Computing, University of Portsmouth, Buckingham Building, Portsmouth PO1 3HE, UK; 5 School of Computing and Mathematical Sciences, Auckland University of Technology, Private Bag 92006, Auckland 1142, New Zealand

## Abstract

**Background:**

NSCLC exhibits considerable heterogeneity in its sensitivity to chemotherapy and similar heterogeneity is noted in vitro in a variety of model systems. This study has tested the hypothesis that the molecular basis of the observed in vitro chemosensitivity of NSCLC lies within the known resistance mechanisms inherent to these patients' tumors.

**Methods:**

The chemosensitivity of a series of 49 NSCLC tumors was assessed using the ATP-based tumor chemosensitivity assay (ATP-TCA) and compared with quantitative expression of resistance genes measured by RT-PCR in a Taqman Array™ following extraction of RNA from formalin-fixed paraffin-embedded (FFPE) tissue.

**Results:**

There was considerable heterogeneity between tumors within the ATP-TCA, and while this showed no direct correlation with individual gene expression, there was strong correlation of multi-gene signatures for many of the single agents and combinations tested. For instance, docetaxel activity showed some dependence on the expression of drug pumps, while cisplatin activity showed some dependence on DNA repair enzyme expression. Activity of both drugs was influenced more strongly still by the expression of anti- and pro-apoptotic genes by the tumor for both docetaxel and cisplatin. The doublet combinations of cisplatin with gemcitabine and cisplatin with docetaxel showed gene expression signatures incorporating resistance mechanisms for both agents.

**Conclusion:**

Genes predicted to be involved in known mechanisms drug sensitivity and resistance correlate well with in vitro chemosensitivity and may allow the definition of predictive signatures to guide individualized chemotherapy in lung cancer.

## Background

The variable response to chemotherapy with platinum containing regimens in NSCLC is well recognized clinically. Patients crossing from one regimen to the alternate in clinical trials commonly show responses [1], suggesting that it might be possible to optimize therapy for individual patients if it was also possible to determine which regimen would be most effective. We and others have used cellular tumor response assays to show similar heterogeneity in NSCLC [2-5], and data from other tumor types such as ovarian cancer suggests that these tests correlate relatively well with outcome [6,7], despite the rapid development of resistance in many patients. However, such tests using primary tumour-derived cells are time-consuming to perform and need a large amount of tumor tissue, far more than it is generally feasible to obtain by bronchoscopic or needle biopsy in most lung cancer patients. This means that there is currently no direct correlation of clinical outcomes with ATP-based tumor chemosensitivity assay (ATP-TCA) results in lung cancer, but there is good evidence to suggest that low passage cell line models or primary tumour-derived cells (as used here) are more reliable predictors of chemosensitivity than cell lines [8].

It has long been a goal of cancer research to produce predictive molecular assays capable of widespread use. Single genes are rarely useful, unless they happen to be the targets of the drugs concerned, but until recently it has been difficult to produce predictive multigene signatures. In practice, there are two possible approaches to the generation of multigene signatures for predictive chemosensitivity testing. The first is to screen very large numbers of genes using hybridization arrays to generate signatures that correlate with clinical outcome [9]. The second approach is hypothesis-driven, using current knowledge of the pathways involved in resistance and sensitivity to individual drugs to generate sets of candidate genes likely to be predictive [10]. We have taken the latter approach and have designed genesets for chemosensitivity prediction based on published information and previous studies. To test this, we have examined their correlation with *in vitro *chemosensitivity data which allows multiple single agents and combinations to be studied, something that would clearly be impossible in patients.

In, the UK cisplatin-containing regimens are recommended for primary chemotherapy of inoperable NSCLC, and at the time we started this study, pemetrexed was not available [11,12]. Of these, the most commonly used in the UK are cisplatin with gemcitabine and cisplatin with docetaxel. Docetaxel is also used as a single agent for patients with reduced tolerance to chemotherapy, post-surgery or on relapse. Patients that respond to one of these regimens may respond to another: such crossover effects provide evidence of heterogeneity of chemosensitivity in NSCLC. Not all NSCLC patients will benefit from the same treatment, and the molecular mechanisms involved are still largely unknown. This study has tested the hypothesis that the molecular basis of this difference lies within the known resistance mechanisms inherent to these patients' tumors. The resistance to anti-cancer drugs involves many mechanisms [13,14], though those mechanisms involved for cisplatin, gemcitabine and taxane resistance have been extensively studied across a wide range of tumors.

Studies of cisplatin resistance in lung cancer are relatively rare, though some data exist. For example increased expression of the DNA repair enzyme *ERCC1 *has been associated with worse outcome in NSCLC patients treated with cisplatin-based chemotherapy [15], and has now been used with some success to predict response to platinum-based chemotherapy [16], though it is unlikely that single markers will be sufficient. The consensus seems to be that cisplatin resistance mechanisms include decreased drug accumulation, enhanced detoxification, and increased DNA repair efficiency [13]. The one exception to this is the presence of mismatch repair defects, which paradoxically increase the toxicity of cisplatin in many tumor types [17-19]. Most membrane transporters, such as p-glycoprotein (*MDR1*) are not involved in cisplatin resistance, but the Copper-transporting ATPase (*ATP7B*) has been implicated in a number of studies [20]. Detoxification of cisplatin includes gluthione conjugation, and glutathione S-transferase (*GSTπ*) has been implicated, as have two members of the multidrug resistance related protein family, MRP1 and MRP3, which are required to transport conjugated xenobiotics [21]. Repair of platinated DNA is primarily by nucleotide excision repair [22]. Expression of *XPA*, *XPG*, *XPF*, *ERCC1 *and *ERCC2 *are apparently involved, but the relative importance of each in this process is uncertain. In addition, the influence of apoptosis related genes has been implicated in resistance to cisplatin, particularly those such as *BCL2 *and *SURVIVIN *which inhibit apoptosis [14,23].

A number of studies of potential gemcitabine resistance mechanisms have been performed in lung cancer using PCR or immunohistochemical methods. Multiple membrane transporters, target enzymes, enzymes involved in the metabolism of gemcitabine and alterations in the apoptotic pathways have been implicated in sensitivity and resistance to this drug in a variety of human tumor types [24]. To date, there is evidence that several genes involved in gemcitabine metabolism, particularly human equilibrative nucleoside transporter 1 (*hENT1*) and cytosolic 5'-nucleotidase type II (*cN-II*), are involved in NSCLC resistance [25]. Resistance has also been linked to the expression of DNA repair genes, particularly *ERCC1*, ribonucleotide reductase subunits 1 and 2 (*RRM1 *and *RRM2*) [26], though immunohistochemical expression of these proteins, RAD51 or BRCA1 could not be shown to have an effect [27]. Recently, multidrug resistance associated protein 5 (*MRP5*), a membrane located pump, has also been implicated in gemcitabine drug resistance [28].

The taxanes stablise microtubules and prevent their turnover, leading to apoptosis in many rapidly dividing cell lines and some tumors. However, they are susceptible to xenobiotic pump mechanisms, including P-glycoprotein (*MDR1*), *MRP*, breast cancer related protein (*BCRP*) and major vault protein (*MVP*). There has been relatively little work on the ability of cells to resist the action of taxanes based on resistance to apoptosis, but several studies suggest that this is likely to be of importance [4,29,30].

It is generally assumed that the same resistance mechanisms used by cells to circumvent single agent activity will also be used against combinations of these drugs. There are few mechanistic studies of resistance to platinum with gemcitabine, or platinum with taxane to confirm or refute this supposition. However, one study by Smith *et al*. [31] in a series of endometrial cell lines suggested that *GSTπ*, *MSH2*, *P53*, and *ERCC1 *may be involved, confirming earlier single agent data implicating these mechanisms [13]. In addition, combining *ERCC1 *and *RRM1 *has been shown to have predictive efficacy for the combination of platinum and gemcitabine [32].

The current study has tested the hypothesis that the molecular basis of the observed difference in sensitivity *in vitro *between primary tumor-derived cells in the ATP-TCA lies within the known resistance mechanisms inherent to these patients' tumor cells. The mechanisms of cellular (i.e. non-pharmacokinetic) resistance to chemotherapy include: down-regulation of target expression, drug metabolism, membrane-located xenobiotic pumps, altered susceptibility to apoptosis, and altered growth/cell cycle or differentiation. Knowledge of these pathways enabled the design of a Taqman Array microfluidic qRT-PCR card to include 92 genes known or hypothesized to be involved in drug resistance/sensitivity to cytotoxic agents, including those described above (table 1). The Chemosensitivity Gene Expression Array (CGEA-1, CanTech Ltd, Portsmouth, UK) also included four housekeeping genes to allow standardisation of the results for comparison of individual tumor data. The CGEA is not comprehensive, but has enabled us to establish the degree to which *in vitro *sensitivity data correlate with gene expression data to determine the likely contribution of individual genes to drug sensitivity and resistance in NSCLC.

**Table 1:** Genes included on Taqman array, classified by their major contribution to drug resistance mechanisms.

Apoptosis	DNA repair	Proliferation	Pumps/Detox
*AKT*	*ATM*	*APC C-term*	*ATP7B*

*APAF1*	*BRCA1*	*APC N-term*	*BCRP*

*BAD*	*ERCC1*	*β-TUBULIN III*	*CES1*

*BAX*	*ERCC2*	*COX2*	*CES2*

*BCL2*	*GTF2H2*	*EGFR*	*cN II*

*BCL-x(L)*	*MGMT*	*HER2*	*DPD*

*BID*	*MLH1*	*HER3*	*FPGS*

*c-FLIP*	*MSH2*	*HER4*	*γ H2AX*

*FAS*	*MSH6*	*HIF1A*	*GCLC*

*FASL*	*RAD51*	*KI67*	*GCLM*

*HSP60*	*TOPO I*	*P16*	*GSTπ*

*HSP70*	*TOPO IIa*	*P21*	*hENT1*

*HSP90*	*TOPO IIb*	*P27*	*hENT2*

*IAP2*	*XPA*	*P53*	*MDR1*

*IGF1*	*XRCC1*	*VEGF*	*MRP1*

*IGF1R*	*XRCC5*		*MRP2*

*IGF2*	*XRCC6*		*MRP3*

*IGF2R*			*MRP4*

*IGFBP1*			*MRP5*

*IGFBP2*			*MRP6*

*MCJ*			*MRP8*

*MCL1*			*MTII*

*mTOR*			*MVP*

*NFkB*	**House-keeping gene**		*OPRT*

*PIK3CA*	*18S*		*RRM1*

*PTEN*	*HPRT*		*SOD1*

*STAT3*	*PBGD*		*TAP1*

*SURVIVIN*	*SDHA*		*TAP2*

*XIAP*	*TBP*		*TAP4*

## Methods

In this study we have used quantitative Reverse Transcriptase Polymerase Chain Reaction (qRT-PCR) to examine the expression of several transporter and metabolism-related genes previously shown by ourselves and others (see above) to be involved in resistance to chemotherapy with cisplatin, gemcitabine or taxanes in NSCLC. In addition we have examined several other mechanisms likely to influence the effectiveness of chemotherapy which have been less commonly studied. The RT-PCR expression profiles obtained from stored formalin-fixed paraffin-embedded tissue have been compared with quantitative in vitro chemosensitivity data obtained for the same tumors using the ATP-based chemosensitivity assay (ATP-TCA).

### Patients and Samples

A total of 62 specimens were obtained from surgical resections of NSCLC tumors from patients with a median age of 69 years, range 54 – 87 years. There were 35 males and 27 females. Of these, 49 fresh samples proved suitable for ATP-TCA. All cases had formalin-fixed paraffin-embedded (FFPE) material taken for diagnostic histology, and gave written informed consent for the study, which received multicentre research ethics committee approval. The FFPE blocks subsequently provided a source of material for qRT-PCR. The breakdown by histological tumor type is given in table 2.

**Table 2:** Patient histological classification in the initial surgical series used to generate signatures.

Description	Number
Squamous cell carcinoma	21

Adenocarcinoma	20

Neuroendocrine NSCLC	3

Poorly differentiated carcinoma	5

Total on study:	49

*Excluded:*	

- Metastatic	4

- Carcinoid	1

- No cells in sample	7

- Contamination of sample/culture	2

- Failure of assay	1

Total	64

### ATP-TCA

In all cases, ATP-TCA data was obtained as previously published [33,34]. Briefly, samples were transported to the laboratory in 25 ml specimen bottles containing cooled transport medium consisting of DMEM (Sigma, Poole, Dorset, UK) with added antibiotics. Tumor cells were obtained by enzymatic dissociation, washed in a serum-free complete assay medium (CAM; DCS, Hamburg, Germany), and purified by density centrifugation to remove debris. The cells were washed and resuspended in CAM for plating in 96 well polypropylene plates at 20,000 cells per well with six dilutions of four drugs or combinations tested in triplicate with two rows of medium only and maximum inhibitor controls. At least 10% neoplastic cells for successful ATP-TCA. The drug concentrations used are given in table 3. The plates were incubated for six days at 37°C with 5% CO_2_, and the remaining ATP extracted with tumor cell extraction reagent (TCER; DCS). Aliquots of the extract were transferred to a white 96 well polystyrene plate to which an equal amount of luciferin-luciferase was added. The resulting luminescence was read in a luminometer (MPLX, Berthold Diagnostic Systems, Germany) and the data transferred to an Excel (Microsoft) spreadsheet for analysis. The results were expressed as the percentage inhibition at each concentration tested, and a summary index representing the sum of the surviving fraction of cells at each dilution tested as 600-Sum(Inh200....Inh6.25) [35], where 0 equals complete inhibition and 600 equals no effect.

**Table 3:** Drug list for ATP-TCA with manufacturer, given with the 100% test drug concentration (TDC) used in the assay, and the number of tumors tested.

Drug or combination	Manufacturer	TDC (μM)	Number tested
Docetaxel	Sanofi-Aventis	3.00	43

Cisplatin	Bristol-Myers-Squibb	10.00	41

Gemcitabine	Eli Lilly	40.04	39

Cisplatin with Gemcitabine	As single agents	As single agents	38

Docetaxel with Cisplatin	As single agents	As single agents	22

Docetaxel with Gemcitabine	As single agents	As single agents	21

Some tumors had too few cells for all agents/combinations to be tested.

### Extraction of RNA from FFPE tumor tissue

Eight 10 μm curl sections were cut from formalin-fixed paraffin-processed blocks of lung including at least 75% tumor tissue, placed in a 1.5 ml microcentrifuge tube and heated at 70°C in a Stuart SBH200D heating block for 20 min to allow excess paraffin wax to be removed from the tissue using a sterile fine tip plastic pasteur pipette. Experiments with lung tissue only showed that this was unlikely to interfere with the assay (data not shown). Pre-warmed xylene (1 ml) was then added to the tube and heated at 50°C for 10 min. The microfuge tube was then removed from the heating block, at centrifuged at 12,000 × g for 2 min in a Sanyo MSE Microcentaur microcentrifuge. Waste xylene was then removed by pipette and the xylene wash was repeated twice more. After the final wash the tissue had uncurled.

Residual xylene was removed by the addition of 1.0 ml of 100% ethanol to the dewaxed tissue sections, which was allowed to stand for 10 min at room temperature. The tissue was then centrifuged 12,000 × g for 5 min and the ethanol removed by pipette, and the process repeated once more with 100% ethanol. The tissue was then rehydrated with 1.0 ml 90% ethanol, for 5 min and finally washed in 1.0 ml 70% ethanol for 5 min. The microfuge lids were then opened to allow the ethanol to evaporate completely prior to protease digestion.

Protease digestion was performed by use of a Recoverall kit™ (Applied Biosystems, AM1975). Digestion buffer (400 μl) and protease (4 μl) were added to each tissue sample and incubated for 3 hours at 50°C, with occasional flicking of the tube to assist uniform digestion. If the tissue was not digested to a clear solution, a further 60 minute incubation was performed, following which 480 μl of the Ambion RecoverAll™ Isolation Additive was added to the microfuge tube, which was then vortex mixed for 20 seconds and allowed to stand for 15 min at room temperature. The tubes were pulse spun in a microfuge at 12,000 × g for 30 seconds before opening to ensure that the liquid was at the bottom of the tube and the lysate was then passed ≥ 5 times through a 0.8 mm needle (21 gauge) fitted to a 1 ml sterile polypropylene syringe to break up any large pieces of tissue. Two 240 μl aliquots of the resulting lysate were then stored at -20°C for RNA extraction.

RNA extraction was performed using the Recoverall kit™. Briefly, the sample lysate was slow thawed at 4°C with gentle vortexing before the addition of 550 μl of 100% ethanol added to each tube. Filter cartridges for RNA isolation were inserted in collection tubes, and 700 μl of the lysate/ethanol mix pipetted onto the centre of each cartridge. The cartridges were centrifuged at 10,000 × g for 60 seconds, the flow through discarded and the filter cartridge re-inserted into the same collection tube. This was repeated twice more until all the lysate had been processed for each sample. The cartridges were washed according to the manufacturer's instructions and the flow through discarded. DNase treatment of the immobilized nucleic acids was performed by the addition of 60 μl of DNase mix to the centre of each Filter Cartridge, incubation for 30 min at room temperature, followed by further washes. Finally, the filter cartridge was placed into a fresh collection tube and 30 μl of heated (95°C) nuclease free water placed into the centre of the filter. Following incubation at room temperature for 60 seconds, the filters were microfuged for 60 seconds at 13,000 rpm (13,000 × g). This was repeated to give a final volume of 60 μl total RNA. Purity and quantity was checked spectrophotometry at 260 nm and 280 nm by placing 1.3 μl of eluate on the sampling pedestal of a scanning NanoDrop ND-1000™ spectrophotometer. Aliquots of each sample were stored at -80°C or reverse transcribed to produce cDNA in a two step RT-PCR reaction.

### Two-step RT-PCR

Reverse transcription was performed using an ABI High-Capacity cDNA Archive Kit (cat 4322171) according to the manufacturer's instructions. Briefly, an aliquot of 75 μl master mix was added to a 0.2 ml PCR tube to which an equal volume of purified RNA diluted in nuclease free water was added. For RT negative wells, a 15 μl master mix was prepared where the multiscibe RT volume was replaced with nuclease-free water. To this was added 15 μl of diluted RNA in nuclease-free water. The final RNA concentration in the RT mix was 50 ng/μl. The tubes were kept in a chilled cooling block until ready to load into the thermal cycler (Hybaid Omn-E). Cycling conditions were step 1, 25°C × 10 min, step 2, 37°C for 120 min. After removal from the thermal cycler, the tubes were pulse spun in a microfuge at 12,000 × g for 30 seconds and stored overnight at 4°C or used immediately. cDNA content was measured using a NanoDrop™ spectrophotometer prior to use in a 'sighting shot' PCR reaction which was performed for all samples to ensure housekeeping gene expression and to confirm that the cDNA was suitable for Taqman Array evaluation.

Pre-Taqman array 'sighting shot' evaluation of newly prepared cDNA was performed by SYBR green PCR for HMBS (PBGD) by adding a 5 μl volumes of 10 μg and 2.5 μg cDNA into one of two wells of a polystyrene PCR plate, each well of which contained 20 μl of master mix (diluted SYBR Green ×2 master mix; Sigma Aldrich S-4438), in nuclease-free water to which primers and MgCl2 had been added. RT negative controls were also included for each sample. PCR was run for 40 cycles in a BioRad i-Cycler and the results transferred to a Microsoft Excel spreadsheet for analysis. Samples with cycle thresholds for detection (Cts) below 35 and undetectable results from the RT negative control were deemed evaluable for use in a Taqman array.

GCEA Taqman arrays were run according to the manufacturer's instructions. Each sample was made up with Taqman ×2 Universal Master Mix and mixed with an equal volume of cDNA to give a final concentration of 300 ng/μl suitable for the small volume dry PCR Taqman array wells. All four samples were then each pipetted into two ports (100 μl per port) of the 384 well card, for the 96 genes arrayed.

The loaded CGEA Taqman array was then placed, port upwards, into a balanced centrifuge (type, address) and spun at 380 × g to fill the card. This was checked and the card spun again at 380 × g to remove any air bubbles. The card was then placed in a Taqman array slide sealer, sealed, and the loading ports cut from the card before it was loaded into an AB 7900HT thermal cycler. PCR was performed for 90 min with the following conditions: AmpErase UNG Activation for 2 min at 50°C; AmpliTaq Gold DNA Polymerase Activation for 10 min at 94.5°C; followed by 40 cycles each of Melt Anneal/Extend for 30 sec at 97°C and 1 min at 59.7°C.

The 'Auto Threshold Cycle' function was performed at the end of the run and resulting Ct data from the CGEA Taqman array card was transferred to a Microsoft Excel spreadsheet, controls checked, and the data transferred to a Microsoft Access database for further analysis.

During the course of the study three standard curve cards were performed using the same cDNA sample with the following final concentrations of cDNA 1100 ng/μl, 300 ng/μl, 150 ng/μl and 75 ng/μl. On examining each of the multiple targets standard curves and primer/probe efficiencies, the optimal final concentration of cDNA was deemed to be 300 ng/μl, and used for all samples. In addition, two replicate cards were run with the same cDNA samples, using three replicates at a final concentration cDNA of 300 ng/μl, and one no template control (NTC; nuclease-free water) on each card.

Ct values were standardised by reference to *HMBS *(*PBGD*), the least variable housekeeping gene of the four present on the array, which was detected at cycle 29, to avoid errors due to differences in efficiency between the HK and test genes which were present from cycle 27 – 35 in most cases. The standard method of analysis (deltaCt) was inappropriate for linear regression as it skews the data obtained, and therefore normalised the data by logarithmic transformation. A logarithmic gene expression ratio (GER) was calculated as ln(2^-Ct [test]^/2^-Ct [PBGD]^) and used for comparison with ATP-TCA data by multiple linear regression using SPSS ver 14.0 and Analyse-It software.

### Data Analysis

Sample size calculations for the in vitro chemosensitivity correlation with the CGEA data were performed using an on-line multiple regression sample size calculator http://www.danielsoper.com/statcalc based on analysis of up to 25 variables (predictors) and an anticipated r-squared of 0.5 with 80% power and alpha 0.05, giving a required sample size of 36 patients for each drug or combination measured.

The data from ATP-TCA, and qRT-PCR studies were collected into an Access database (Microsoft), from which descriptive statistics were generated. Statistical comparison of ATP-TCA results for individual drugs (continuous dependent variable) with CGEA gene expression data was performed by multiple linear regression with forward selection of variables using SPSS ver 14.0 (SPSS Inc, Chicago, USA). For each variable, inclusion was dependent upon a probability of F > 0.1, i.e. the threshold for inclusion of a gene into the forward linear regression model within SPSS, based on initial assessment of the most appropriate model size. The PRESS (prediction residual sum of squares) graphs shown use an adjusted regression method employed to prevent overfitting, as a 'leave one out method'. No intercept was term was included, and genes were added by forward regression according to their univariate correlations following entry of each gene. The number of genes accepted into each model was based on initial assessment of the most appropriate model size using Akaike Information Criterion versus model size (data not shown). The Akaike Information Criterion (AIC) is a function of model error and size which penalises large models, and the lowest number is regarded as best.

## Results

### ATP-TCA

There were sufficient cells for testing of one drug or more, with evaluable results, in 52 specimens with a diagnosis of NSCLC following surgery and sufficient cells for testing. Three specimens failed to produce results in the assay: one due to low ATP counts at the end of the assay, while the other cultures became infected, probably due to low level contamination within the samples.

**Figure 1:**
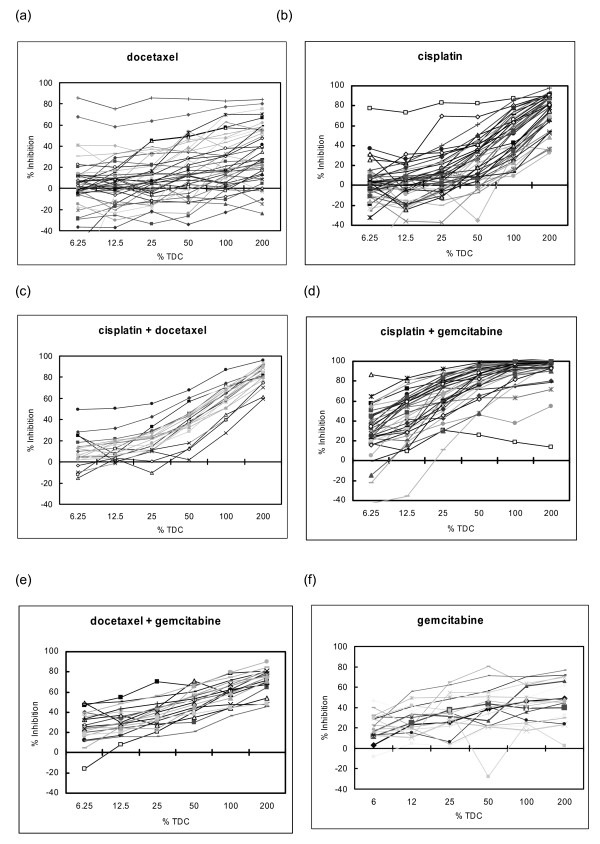
**Heterogeneity of chemosensitivity for (a) docetaxel (b) cisplatin, (c) cisplatin with docetaxel, (d) cisplatin with gemcitabine, and (e) docetaxel with gemcitabine, (f) gemcitabine**. Each line represents a different tumor tested at six concentrations.

The results from the 49 samples with evaluable ATP-TCA data show considerable heterogeneity of chemosensitivity between tumors (figure 1) tested with single agents and combinations. Cisplatin is usually more active than docetaxel, with a steeper concentration-inhibition curve (figure 1). Of the combinations tested, cisplatin with gemcitabine was the most active in this series of experiments (figure 1), and proved more effective than cisplatin with docetaxel in the 21 tumors tested with both combinations (Wilcoxon, p < 0.0001). Several experimental regimens were tested. Comparison of the largest histological sub-groups of NSCLC (i.e. those showing squamous or glandular differentiation) showed no difference in the effect of treatment, and no gender effects were noted on sensitivity to cisplatin, docetaxel or doxorubicin.

**Figure 2:**
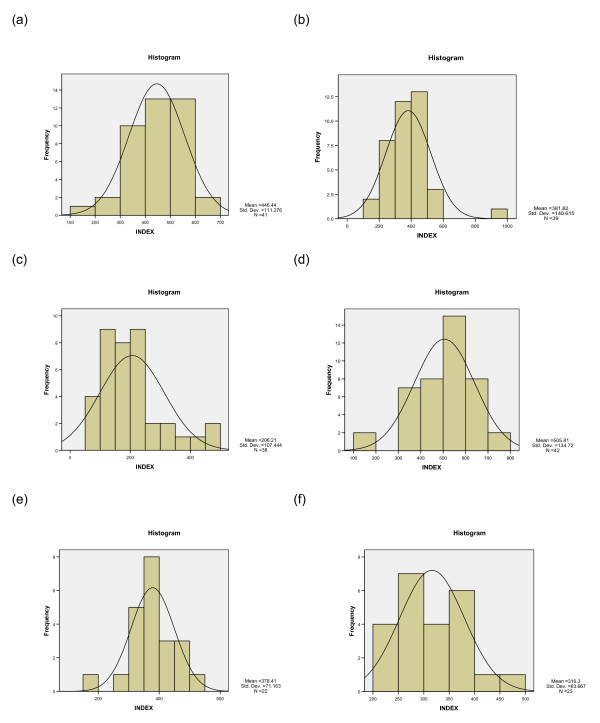
**ATP-TCA results expressed as histograms for IndexSUM for the drugs and combinations most commonly used in NSCLC**. (a) cisplatin (b) gemcitabine (c) cisplatin with gemcitabine (d) docetaxel, (e) docetaxel with cisplatin, (f) docetaxel with gemcitabine.

The ATP-TCA IndexSUM is a natural logarithmic index, ranging from 0 – 600 for inhibition, with zero corresponding to complete cell kill, and 600 equating to no effect. Examination of frequency histograms and IndexSUM data (figure 2, table 4) for each of the drugs tested shows considerable heterogeneity of chemosensitivity between individual tumors for all drugs tested, with the greatest activity (i.e. lowest IndexSUM) for cisplatin with gemcitabine, and cisplatin with docetaxel. The single agents alone are relatively inactive (figure 2) in comparison with the combinations.

**Table 4:** Data for each drug and combination tested, showing median and interquartile range (in brackets) for IC50, IC90 (both expressed as % TDC) and Index_SUM_.

Drug/Combination	IC90 (%TDC)	IC50 (%TDC)	Index_SUM_	Number with > 95% inhibition
Docetaxel	433 297 – 778.5	246 71 – 438	523 419 – 591	0/43

Cisplatin	229 204 – 278	95 71 – 156	466 378 – 513	0/41

Gemcitabine	300 242 – 383	82 22 – 213	378 293 – 446	0/39

Cisplatin with Gemcitabine	80 46 – 143	13 8 – 23	186 136 – 231	19/38

Docetaxel with Cisplatin	213 203 – 231	75 59 – 93	378 339 – 405	0/22

Docetaxel with Gemcitabine	242 224 – 267	45 37 – 78	310 290 – 370	0/21

The final column shows the number of tumors tested and those achieving 95% inhibition at the 100% TDC of those tested for each drug or combination.

**Figure 3:**
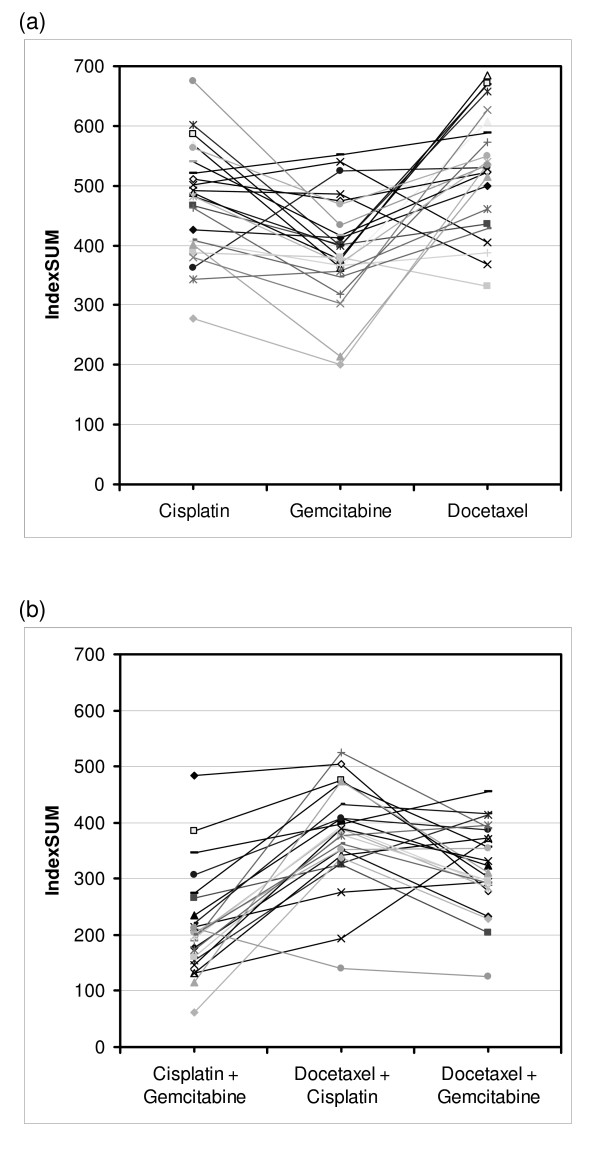
**Cross resistance in the ATP-TCA between (a) drugs and (b) combinations, expressed as IndexSUM**.

All six agents were tested in 24 tumours. Comparison of these paired results by IndexSUM (figure 3) shows the greater sensitivity of NSCLC to gemicitabine with respect to cisplatin (p < 0.001), and docetaxel (p < 0.001). Cisplatin was marginally more active than docetaxel (p < 0.022). However, in 3/24 tumors, cisplatin was the most active agent, while in 3/24 tumors docetaxel was the most active agent, and in 18/24 tumors gemcitabine was the most active agent. Comparison of the three combinations tested showed that cisplatin + gemcitabine was more active than cisplatin + docetaxel (p < 0.001), and docetaxel + gemcitabine (p < 0.001) though the of the two docetaxel containing regimens docetaxel ± gemcitabine was the more active (p < 0.007). However, there was some heterogeneity, though in most (20/24) tumors cisplatin + gemcitabine was the most active combination, in 4/24 tumors docetaxel + gemcitabine was the most active. Either were always better than cisplatin + docetaxel in this series (figure 3, table 4). ATP-TCA data can be used to generate data on additive or synergistic effects between drugs, but this analysis has not been preformed as it is of limited relevance to this paper.

### qRT-PCR

Despite the use of FFPE tissue, all samples were regarded as evaluable on the basis of the housekeeping gene Ct levels (i.e. *PBGD *Ct > 35 cycles). Variation in HK gene levels was limited, with a normal distribution of Ct levels (*PBGD*, mean 29.301 (95% CI 28.630 – 29.971). Ct levels within the detectable range were present for most of the genes present on the Taqman array [see Additional file 1], though some were more rarely expressed than others, and NTC remained undetectable throughout the study.

The two replicate plates showed an intra-assay variation (CoV) of 0.32% and 0.07% on Ct values from the same sample, while the same sample tested five times in different plates showed an inter-assay variation (CoV) of 0.51% for *HMBS*. Two dilution plates were run during the series of Taqman arrays to assess the efficiency of the genes included on the plate. These results show 99% efficiency for *HMBS*.

### Correlation of mechanisms with ATP-TCA data

**Figure 4:**
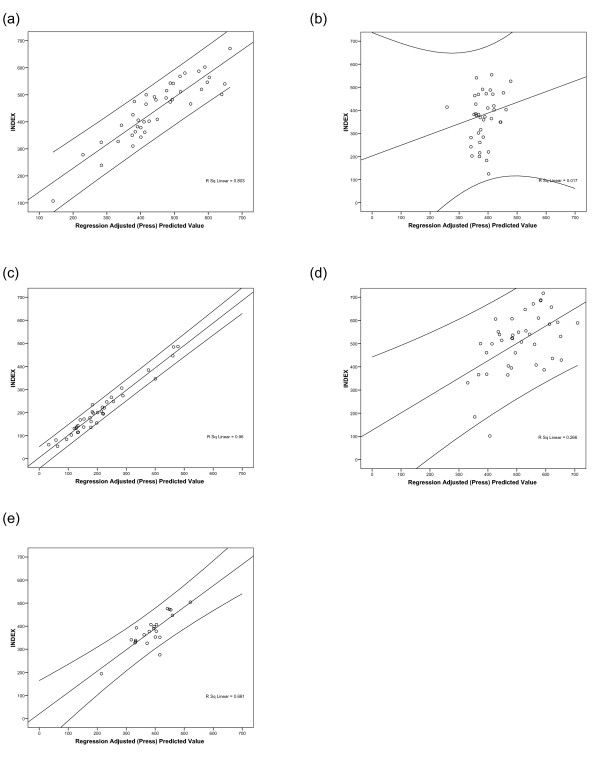
**Multiple linear regression analysis (PRESS) by unsupervised forward selection for (a) cisplatin; (b) gemcitabine; (c) cisplatin with gemcitabine; (d) docetaxel; (e) docetaxel with cisplatin**. No correlation was obtained for docetaxel with gemcitabine.

In general, comparison with genesets linked to particular resistance mechanisms showed good correlation with drugs susceptible to these mechanisms in multiple regression analysis (figure 4), provided that the drugs were active and showed heterogeneity of chemosensitivity with a spread of sensitive and resistant tumors. Although the genes chosen were not a naïve dataset, as all had been related to drug resistance or sensitivity, for the purpose of this report the SPSS analysis included all genes present on the card. Forward multiple regression models were identified for each drug or combination included in the study. The genes involved are shown in table 5, in order of greatest contribution to the model, and the coefficients for each in table 6.

**Table 5:** Genes found to correlate in multivariate linear regression analysis using forward selection: model summaries.

*Model*	*R*	*R*^2^	*Adj R*^2^	*Std. Error*	*Significance* *(ANOVA)*
Cisplatin	0.965	0.931	0.892	36.976	0.001
Gemcitabine	0.277	0.076	0.051	136.326	0.093
Docetaxel	0.664	0.441	0.361	107.579	0.001
Cisplatin with Gemcitabine	0.997	0.995	0.987	12.565	0.001
Cisplatin with Docetaxel	0.959	0.92	0.885	23.375	0.001

**Table 6:** Genes found to correlate in multivariate linear regression analysis using forward selection: coefficients for each gene included in the model by drug or combination tested.

	Unstandardized		Standardized	t	Collinearity Statistics	
Gene	B	Std. Error	Beta	Lower Bound	B	Std. Error
***Cisplatin***						
(Constant)	403.123	33.393		12.072		
*GTF2H2*	64.781	16.558	0.573	3.912	0.129	7.725
*IAP2*	-153.15	16.813	-2.122	-9.109	0.051	19.571
*MRP5*	71.075	8.946	0.799	7.945	0.274	3.651
*P53*	-91.289	11.062	-0.63	-8.252	0.476	2.101
*ATM kinase*	105.764	14.77	1.494	7.161	0.064	15.71
*FPGS*	30.449	9.411	0.391	3.235	0.19	5.263
*mTOR*	-14.902	14.509	-0.146	-1.027	0.137	7.273
*TS*	42.031	7.318	0.584	5.744	0.269	3.724
*MCJ*	92.954	14.616	0.908	6.36	0.136	7.355
*BID*	-49.393	12.785	-0.402	-3.863	0.256	3.9
*Topo I*	-72.192	17.437	-0.688	-4.14	0.1	9.957
*Mcl-1*	57.031	12.755	0.69	4.471	0.116	8.597
*HER2*	-12.172	5.364	-0.179	-2.269	0.448	2.233
*MVP*	-30.265	13.535	-0.197	-2.236	0.357	2.805
						
***Gemcitabine***						
(Constant)	338.553	35.306		9.589		
*MRP4*	-28.047	16.241	-0.277	-1.727	1	1
						
***Docetaxel***						
(Constant)	778.346	103.411		7.527		
*MRP5*	62.715	14.617	0.623	4.291	0.757	1.322
*GSTπ*	-77.649	24.429	-0.461	-3.179	0.76	1.316
*IGFBP2*	-25.582	12.653	-0.265	-2.022	0.933	1.072
*IGFBP1*	28.488	10.056	0.411	2.833	0.76	1.316
*MRP8*	-22.098	9.513	-0.333	-2.323	0.777	1.287
						
***Cisplatin with Gemcitabine***						
(Constant)	350.892	41.063		8.545		
*BAX*	51.989	11.961	0.273	4.346	0.094	10.64
*HER3*	-10.321	4.863	-0.098	-2.122	0.172	5.813
*MRP3*	-19.569	1.948	-0.371	-10.044	0.272	3.681
*AKT*	-150.08	7.205	-1.064	-20.831	0.142	7.063
*MRP8*	26.222	2.484	0.45	10.556	0.203	4.915
*MRP5*	43.708	3.634	0.474	12.026	0.238	4.209
*BCL-x(L)*	178.422	11.378	1.177	15.682	0.066	15.23
*EGFR*	-46.006	4.356	-0.47	-10.562	0.187	5.353
*hENT2*	32.558	4.365	0.427	7.458	0.113	8.855
*XRCC5*	90.76	6.702	0.547	13.543	0.227	4.412
*IGFBP1*	-5.027	2.315	-0.086	-2.171	0.235	4.26
*cN II*	-73.932	9.164	-0.562	-8.067	0.076	13.146
*HPRT*	49.176	4.955	0.715	9.925	0.071	14.029
*RRM1*	-69.228	7.483	-0.63	-9.251	0.08	12.527
*P16*	7.663	2.017	0.146	3.798	0.251	3.989
*TAP2*	-43.744	6.008	-0.284	-7.281	0.243	4.115
*MGMT*	45.697	6.513	0.377	7.016	0.128	7.799
*ATP7B*	-7.418	2.019	-0.14	-3.675	0.255	3.928
*KI67*	-8.882	2.763	-0.161	-3.215	0.147	6.807
*TOPO IIa*	-6.788	1.708	-0.137	-3.973	0.311	3.218
*BCRP*	13.738	3.335	0.191	4.12	0.172	5.8
*VEGF*	12.683	5.573	0.098	2.276	0.199	5.034
						
***Cisplatin with Docetaxel***						
(Constant)	178.403	46.289		3.854		
*APAF1*	38.159	5.752	0.855	6.635	0.345	2.899
*MLH1*	-63.725	6.467	-1.339	-9.854	0.31	3.223
*PTEN*	-20.681	3.038	-0.689	-6.808	0.56	1.787
*GSTπ*	25.097	10.451	0.224	2.401	0.657	1.523
*ATP7B*	10.975	4.529	0.332	2.423	0.306	3.269
*BCL-x(L)*	24.707	11.05	0.25	2.236	0.459	2.177

Cisplatin activity showed strong correlation with resistance gene expression, particularly those genes involved in DNA repair (e.g. *ATM *kinase and *GTF2H2*) and apoptosis (e.g. *IAP2*, *BID*, *MCJ*, *P53*, *mTOR*, and *MCL1*). Few drug pumps were present in the model, though *MRP5 *and *MVP *were present, as well as two nucleotide metabolism genes, *TS *and *FPGS*. Docetaxel activity showed greater correlation with drug pump expression and detoxification mechanisms, with no relationship to DNA repair. Docetaxel showed little activity as a single agent, but was correlated with two MDR-related pumps (*MRP5 *and *MRP8*), and with *GSTπ *expression, as well as IGF binding protein (*IGFBP*). Forced addition of *β-TUBULIN III *to the final model added no benefit to the model. Few of the genes thought to be involved in gemcitabine expression were present on the card, and only *MRP4 *was selected by statistical analysis, though this had a very low level of correlation with gemcitabine sensitivity (figure 4).

Greater levels of correlation were found between gene expression and chemosensitivity to combinations. Cisplatin with gemcitabine showed excellent correlation with a model containing 22 genes, including a number of DNA repair and apoptosis genes, though no less than 4 drug pumps are also included in this model. There were fewer observations for cisplatin with docetaxel due to the order in which drugs were tested in the ATP-TCA: some tumors contained too few cells to test all drugs. Both lists include a number of genes involved in apoptosis, as well as GSTπ and drug pump molecules, together with a several genes involved in DNA repair. It is noted that the first two genes in the cisplatin with docetaxel model are *APAF1*, involved in apoptosis, and *MLH1*, a mismatch repair gene.

No tumor type-specific differences were found between adenocarcinomas and squamous cell carcinomas for any of the drugs studied.

## Discussion

Use of the ATP-TCA has allowed us to examine both single and combination effects for the same tumors, and for the first time to compare chemosensitivity data with the expression of a large number of potential resistance mechanisms using a robust qRT-PCR approach with mRNA obtained from FFPE biopsy material. While the ATP-TCA requires large surgical biopsy specimens, the qRT-PCR method can be performed with a few nanograms of RNA extracted from FFPE tissue. Similar RT-PCR results to these can be produced from the much smaller samples obtained by bronchoscopic or needle biopsy (Gabriel et al., unpublished). If this approach proves to be related to clinical outcome, it may be possible to design tests to predict the efficacy of the two main chemotherapy regimens currently in use for NSCLC treatment and to optimize patient treatment on this basis.

The comparison of quantitative data from the ATP-TCA (CoV 15%) with that from qRT-PCR (CoV 2%) has the advantage that relatively small numbers of tumors are required to obtain data on the genes relevant to resistance and sensitivity to drugs tested in the assay. This may prove to be particularly useful to investigate the mechanisms of sensitivity and resistance for drugs which are rarely used as single agents in specific tumor types, and for new drugs which have not yet entered the clinic. However, for single agents, there is less sensitivity, and less variation between patients in the ATP-TCA, and therefore lower levels of correlation with gene expression were found for the single agents.

The Taqman array card included all of the genes described in the introduction to this paper, but was manufactured before some more recent papers were published which suggest that others may also be important. For instance, for gemcitabine, other genes involved include human concentrative nucleoside transporter (*hCNT3*), deoxycitidine kinase (*dCK*), cytidine deaminase (*CDA*), Cytidine deaminase (*CDD*) and 5'-nucleotidase (*5NT*) gene polymorphisms and *CDD*, *5NT*, deoxycytidine kinase (*DCK*) and *MRP5 *[28,36]. Further work is therefore required to define genesets that might be clinically useful.

The genes identified in this study fall into several categories, linked with much studied mechanisms such as metabolism within the cell, membrane drug pumps, and DNA repair, but also with apoptosis, suggesting that the general susceptibility of the cell to undergo this process may be an important determinant of tumor chemosensitivity, outweighing more specific mechanisms [14]. The genes found to be important by unsupervised forward selection multiple regression match well with those thought to be important from previous studies in cell lines and multiple tumor types. Other statistical methods have been applied (data not shown), including analysis of principal components, cluster analysis and supervised regression models. All show a similar ability to derive models which describe the data, and the importance of genes related to particular resistance/sensitivity mechanisms seem to hold up remarkably well. Inevitably there are some discrepancies between the published literature and our findings, but the number of tumors is as yet relatively small and it is important not to read too much into the individual genes within the models.

Most combinations used in lung cancer have been derived empirically from phase II and phase III clinical trials. However, synergy has been observed in cell lines between cisplatin and several agents: notably taxanes and gemcitabine. The basis of this synergy is unclear for platinum with docetaxel, but is likely to be indirect as platinum is a DNA-damaging agent, while taxanes stabilize microtubules. In contrast, there several groups have shown that gemcitabine can reverse resistance to cisplatin by its inhibition of DNA repair [24,37]. Gemcitabine is only incorporated during DNA replication or repair. Since most solid tumors have a relatively low S-phase fraction, single agent gemcitabine has limited activity (though continuous administration may be cytostatic [38]. Concomitant administration of a DNA damaging agent, such as platinum, leading to upregulated DNA repair will have two effects. Firstly, gemcitabine will be incorporated more readily into DNA of non-dividing cells, and secondly, its ability to inhibit DNA polymerase will tend to prevent resistance to the DNA-damaging agent [39]. The effect on DNA repair seems to occur at much lower concentrations of gemcitabine than direct incorporation, suggesting that there is no need to use high doses of gemcitabine in platinum with gemcitabine combinations. This explains the potency of this regimen in NSCLC, but clearly resistant patients exist, and indeed some tumors in this study were more sensitive to alternative regimens.

While reverse resistance can help to explain why there are so few genes in common when cisplatin and gemcitibane (*MRP5*, *MCL1*, *MVP*) and cisplatin and docetaxel (*GSTπ*, *MRP8*, *FPGS*, *P53*, *DPD*, *IAP2*) are used together as opposed to separately (Table 6), the number of extra genes correlated when cisplatin is used in combination with gemcitabine (n = 19) and docetaxel (n = 14) indicates that there may be still unknown linkages and pathways between NSCLC-related genes that need further exploration.

One of the main reasons behind our decision to study NSCLC first was that there are two common types with very different histopathology and oncogenesis. The finding that there is no difference between the adenocarcinomas and squamous cell carcinomas in this series suggests that resistance and sensitivity to cytotoxic agents, at least, is determined by the gene expression profile of the cell for a particular drug, and that tumor type is of lesser importance. This fits with current clinical practice in which drugs such as cisplatin, docetaxel, and gemcitabine are used for a wide range of different solid tumor types, albeit with varying success.

## Conclusion

In conclusion, the data presented here support the hypothesis that the molecular basis of the observed difference in sensitivity between NSCLC tumors lies within the known resistance mechanisms inherent to these patients' tumors. It suggests that the Taqman array is ideally suited to investigate the presence of these mechanisms in lung tumors alongside cellular chemosensitivity testing with individual drugs or combinations, and this may be of particular relevance to decision making during drug development.

## Competing interests

IAC is a director of CanTech Ltd. The remaining authors declare that they have no competing interests.

## Authors' contributions

IAC, DY, PEA, TJG and AN conceived and designed the study. SG, LAK, MEP, ZG and KP participated in the ATP-TCA studies. FGG, PJ, MEP, LAK, and KP carried out the qRT-PCR studies. Clinical aspects of the study including recruitment, ethics, consent, planning and analysis involved DY, MG, PM, JR, KA, IAC and TJG. Histopathology was performed by MES, BA and IAC. IAC, AN and MP participated in the statistical analysis. All authors participated in the data analysis, drafting of the manuscript and read and approved the final version.

## Pre-publication history

The pre-publication history for this paper can be accessed here:

http://www.biomedcentral.com/1471-2407/9/300/prepub

## Supplementary Material

Additional file 1 The Taqman array. Click here for file
