# Supplementary material for: Resistance gene expression determines the in vitro chemosensitivity of non-small cell lung cancer (NSCLC)
Source: BMC Cancer. 2009 Aug 27;9:300. doi: 10.1186/1471-2407-9-300 (PMC2739227; doi:10.1186/1471-2407-9-300)
Supplement: Additional file 1 — The Taqman array. [file 1471-2407-9-300-S1.pdf]

Tumour number	Sex	Age	Tumour Diagnosis	Cisplatin IndexSUM	Gemcitabine IndexSUM	Docetaxel IndexSUM	Cisplatin + Gemcitabine IndexSUM	Docetaxel + Cisplatin IndexSUM	Docetaxel + Gemcitabine IndexSUM	18s	Akt	APAF1	APC C-term	APC N-term	ATM kinase	ATP7B	Bad	Bax	Bcl-x(L)
										**NR_003286**	**NM_005163**	**NM_181861**	**NM_000038**	**NM_000038**	**NM_000051**	**NM_000053**	**NM_004322**	**NM_004324**	**NM_000633**
1	F	56	Lung Ca Adeno	Undetermined	Undetermined	368	103	Undetermined	Undetermined	26.714071	30.409546	35.263317	35.61232	30.932293	32.68809	34.9393	33.507336	31.261356	30.964888
2	F	53	Lung Ca NSCLC neuroendocrine	Undetermined	Undetermined	Undetermined	Undetermined	Undetermined	497	22.94818	27.002556	30.917788	30.939215	28.549982	30.02757	30.154339	30.885082	26.387508	27.4518
3	F	71	Lung Ca Adeno	Undetermined	Undetermined	Undetermined	Undetermined	Undetermined	548	25.95213	31.840782	35.154106	Undetermined	31.694975	31.757391	Undetermined	36.98911	32.20056	30.949198
4	M	60	Lung Ca SCC	602	400	657	155	328	413	16.268642	26.50678	31.351046	31.692806	28.51433	29.814241	28.927946	30.282375	26.842173	26.399696
5	M	76	Lung Ca Adeno	Undetermined	Undetermined	Undetermined	Undetermined	Undetermined	592	25.418726	28.122845	33.922676	36.98203	31.970816	32.96515	33.911324	32.30986	29.12078	28.455183
6	M	72	Lung Ca Adeno	361	470	184	143	Undetermined	Undetermined	16.614735	25.31822	30.57125	32.117065	27.933676	28.958595	29.956148	29.674833	26.655926	25.95016
7	M	66	Lung Ca NSCLC neuroendocrine	546		500	446	Undetermined	Undetermined	20.369766	27.411245	31.32856	35.90305	29.651726	31.644878	29.704283	31.971111	26.951984	27.449327
8	F	66	Lung Ca Adeno	400	282	539	114	Undetermined	Undetermined	20.97774	27.946842	32.946003	35.518833	30.327417	31.770723	31.872139	33.762123	28.040178	28.270916
9	M	53	Lung Ca Adeno	324	280	395	223	Undetermined	Undetermined	17.776785	26.212011	31.7038	34.37176	29.962687	31.347034	33.66014	29.613209	27.240753	25.79754
10	M	60	Lung Ca SCC	239	259	102	168	Undetermined	Undetermined	20.53172	27.834936	33.66372	36.898014	30.919922	33.95427	32.716274	32.03238	28.244175	27.564842
11	M	68	Lung Ca Adeno	475	300	647	196	333	Undetermined	18.697548	26.96104	33.787106	36.129192	30.46932	33.21678	31.921825	30.927626	27.363165	26.401237
12	F	57	Lung Ca SCC	542	Undetermined	365	Undetermined	Undetermined	Undetermined	28.988285	33.840485	35.093117	33.973206	31.181301	32.95219	Undetermined	35.791477	31.711935	31.754454
13	M	69	Lung Ca Adeno	363	524	531	306	407	388	17.983992	26.457954	30.951214	31.687496	27.363588	29.810476	28.894907	29.962772	25.957472	24.94512
14	F	69	Lung Ca Adeno	500	383	610	220	Undetermined	Undetermined	27.93206	31.68249	34.69092	36.96332	31.925821	34.46617	Undetermined	34.971886	31.433228	29.927082
15	F	65	Lung Ca SCC	350	314	507	138	Undetermined	Undetermined	24.196888	30.96267	36.956978	34.30382	31.539827	31.959967	32.868164	33.581356	31.272964	30.229836
16	M	56	Lung Ca SCC	540	417	522	273	471	357	22.877743	29.578022	36.593163	Undetermined	Undetermined	Undetermined	Undetermined	33.36884	30.945957	30.594805
17	F	69	Lung Ca SCC	473	240	763	248	447		19.234324	26.82773	31.264515	32.429306	28.217466	30.624058	30.948982	30.718983	26.666431	26.27496
18	F	66	Lung Ca SCC	511	474	523	484	504	278	20.90831	29.817995	31.942081	34.51961	29.953047	29.946459	29.970993	31.842485	27.219213	27.658463
19	F	59	Lung Ca SCC	587	380	671	384	476	303	23.006903	29.891693	33.159573	36.908722	29.681583	30.12098	31.92443	32.501137	27.64781	28.497347
20	F	60	Lung Ca (Bronchogenic Ca)	107	181		54	Undetermined	Undetermined	24.72551	31.972815	35.977642	Undetermined	32.30086	33.850716	35.930424	35.95356	30.963612	31.476103
21	M	71	Lung Ca	671	489	688	486	Undetermined	Undetermined	17.298313	26.910837	33.052643	35.44271	28.955404	32.483868	31.348434	31.048216	27.794329	27.950775
22	M	56	Lung Ca Adeno	Undetermined	Undetermined	Undetermined	Undetermined	Undetermined	Undetermined	25.540216	32.097683	35.27648	Undetermined	33.929886	32.89979	33.929096	35.67755	31.95862	32.195038
23	M	60	Lung Ca SCC	310	467	555	206	Undetermined	Undetermined	21.549131	28.947092	31.887089	33.122585	28.942732	30.341991	Undetermined	30.968756	26.95011	27.946156
24	F	63	Lung Ca SCC	488	376	685	132	341	372	17.059437	25.956457	33.36124	Undetermined	30.391266	32.236496	29.92599	28.88511	25.445257	26.93253
25	M	71	Lung Ca Adeno	327	198		84	Undetermined	Undetermined	15.974095	24.520163	31.530624	35.90667	29.440872	30.947157	29.050295	27.857393	24.954105	25.740435
26	M	72	Lung Ca Adeno	501	539	404	131	194	370	28.375862	29.973867	Undetermined	Undetermined	31.768545	31.970493	Undetermined	32.91848	28.938768	28.967909
27	F	62	Lung Ca SCC	482	370	540	198	390	284	14.944512	26.4403	31.39722	33.162113	28.311949	30.056639	29.528978	28.58903	25.4826	26.555979
28	M	68	Lung Ca Adeno	564	468	549	203	352	355	14.780874	25.190474	29.408033	31.539536	27.25208	27.79518	28.371836	28.272179	24.7261	25.941038
29	M	79	Lung Ca SCC	406	367	387	194	393	290	16.995415	25.960274	31.957848	34.63838	28.949516	29.599731	31.932787	28.98156	25.062855	26.820597
30	M	70	Lung Ca Adeno	543	123	584	81	Undetermined	Undetermined	14.937568	26.003815	Undetermined	32.89504	28.950815	28.502485	26.627508	28.934431	25.897005	25.612095
31	F	61	Lung Ca SCC	409	347	429	200	363	292	17.964664	26.01144	32.97061	36.64163	31.906431	34.267574	31.02158	29.528479	26.954674	25.75518
32	M	65	Lung Ca SCC	520	552	589	346	398	454	15.482352	26.80378	32.903225	34.18976	30.6214	32.831375	30.23239	29.397684	26.513182	26.110115
33	M	71	Lung Ca SCC	382	347	461	137	Undetermined	Undetermined	21.757376	28.96009	34.968197	35.93673	32.73913	34.410866	33.355118	32.075382	29.646906	29.678087
34	F	55	Lung Ca Adeno	378	218	366	Undetermined	Undetermined	Undetermined	24.931095	30.99979	34.818672	36.93956	31.734177	31.949518	32.92736	33.87342	30.58395	30.378006
35	M	82	Lung Ca SCC	278	200	536	61	338	229	15.9835415	25.539358	31.981548	33.920002	28.911745	30.39781	30.804699	29.696632	24.945831	25.439281
36	F	67	Lung Ca Adeno	387	380	331	161	378	296	23.886917	28.540384	Undetermined	Undetermined	36.92527	Undetermined	Undetermined	32.970905	29.736147	29.950542
37	M	56	Lung Ca Atypical/ Neuroendocrine	580	425	408	Undetermined	Undetermined	Undetermined	17.95884	27.939823	30.872807	31.918232	27.921362	28.691822	27.293121	31.118885	26.98192	27.62604
38	M	63	Lung Ca Adeno	402	214	514	115	473	310	27.807209	30.881313	34.568455	Undetermined	31.611538	33.274693	32.60729	35.993202	29.74717	29.94028
39	M	63	Lung Ca SCC	380	302	627	146	390	332	20.086416	27.706059	36.95478	Undetermined	35.698463	35.951324	35.001736	31.466597	28.79096	28.687721
40	M	60	Lung Ca Adeno	515		551	Undetermined	Undetermined	Undetermined	27.45663	31.736649	35.95672	Undetermined	32.936954	32.963528	35.974792	35.013737	29.54416	31.283836
41	M	80	Lung Ca SCC	343	357	460	172	377	395	17.78681	26.328468	30.696932	31.534758	27.913458	28.965961	31.956636	30.475624	25.454235	26.868826
42	M	65	Lung Ca SCC	465	462	606	246	Undetermined	Undetermined	18.787128	28.95673	34.973766	Undetermined	32.652622	33.81961	33.802605	32.951015	28.667955	29.577133

Bcl2	BCRP	Beta-tubulin III	Bid	BRCA1	c-FLIP	CES1	CES2	cN II	COX2	DPD	EGFR	ERCC1	ERCC2	Fas	Fas-L	FPGS	gamma H2AX
**NM_138578**	**NM_004827**	**NM_006086**	**NM_197966**	**NM_007294**	**NM_001025195**	**NM_003869**	**NM_003879**	**NM_012229**	**NM_000963**	**NM_000110**	**NM_005228**	**NM_001983**	**NM_000400**	**NM_000043**	**NM_000639**	**NM_004957**	**NM_002105**
34.907368	34.431854	34.720512	33.828964	Undetermined	30.54639	31.952179	35.12412	31.364147	27.332514	29.949503	29.518114	29.957592	29.892218	32.991898	Undetermined	34.973156	29.831917
29.307116	32.879787	29.94584	28.965998	29.79718	27.729446	29.454796	30.699253	28.740969	30.00571	28.353333	30.989481	27.597687	29.360344	30.531555	Undetermined	32.77168	26.993097
36.9343	Undetermined	31.345772	35.960495	36.9917	31.95329	33.96483	34.98945	32.437393	30.002525	32.975372	28.972416	30.943834	30.921886	33.58876	Undetermined	31.75639	26.770266
30.981188	28.376247	30.499887	27.888191	29.476065	28.127993	22.962276	30.974506	26.099571	32.392612	28.942965	27.291126	28.206783	30.38691	28.930668	32.915104	30.212593	25.973055
33.64345	32.410667	33.969982	32.07665	34.432278	29.109806	30.744658	33.410454	30.308193	31.966902	28.623983	30.00562	29.954044	33.954697	32.14229	35.93602	34.947235	28.98218
29.862091	30.336266	29.798199	28.127935	30.581123	25.944786	27.422836	28.901333	25.911293	30.943872	27.957994	27.97012	27.328274	29.961025	28.840523	32.616695	27.997469	27.071772
29.414986	32.436665	29.276161	31.679144	29.947882	28.27771	31.121109	31.769526	28.66053	32.896305	29.929924	29.620804	28.478848	30.58619	31.950863	36.967777	32.9587	24.851044
33.938038	33.935616	30.962057	31.992441	34.843506	28.812565	31.09815	32.751625	28.015032	33.67293	29.935678	30.687555	29.940825	32.71112	31.801369	33.114143	32.720158	26.989313
31.31841	33.31438	26.97744	30.294159	31.20366	26.948399	28.900612	30.755394	26.563745	28.993704	28.922964	27.96725	27.823933	29.678556	28.623598	33.7688	30.952381	27.842188
32.719337	33.945915	29.603363	30.965988	31.832533	28.300692	30.937	30.73805	28.334538	31.36909	30.70882	27.776365	29.191854	31.58944	29.920874	35.957623	35.108597	27.00875
30.83625	32.52032	30.838724	30.580431	32.93058	27.564276	28.973541	31.632626	27.963726	29.68512	29.350168	28.955633	27.959942	29.932932	28.688164	33.630165	31.516296	27.96209
Undetermined	Undetermined	31.709864	33.97341	35.317444	32.34367	33.638985	34.98752	32.550316	32.47929	33.19299	28.694897	33.793682	32.522057	34.619984	Undetermined	35.446293	28.23525
30.947966	31.64682	29.96659	28.677628	31.448284	26.136137	27.305902	29.993607	25.936466	28.957922	26.197874	28.73199	27.844755	29.936872	28.596828	36.631645	29.897327	28.017242
Undetermined	33.812733	32.38548	33.712654	34.136227	29.957253	31.951056	35.96483	31.946934	31.980598	30.9314	31.918474	32.889008	34.947647	33.685574	Undetermined	36.425175	31.96961
34.95895	35.480534	35.954655	33.98521	33.148735	29.755959	30.598019	33.11458	30.802994	31.569124	31.503372	32.818394	31.260315	33.159298	32.924362	Undetermined	33.497215	31.991068
32.718613	35.933563	34.867916	33.30204	36.99408	32.204002	33.00261	32.49753	31.624025	35.298428	36.753147	31.978523	29.944284	31.691965	31.614296	Undetermined	35.95934	27.756966
30.845087	30.359503	28.725435	28.977282	29.828749	27.12932	28.253668	29.30714	26.60189	29.38602	28.095888	26.624329	27.516033	29.131027	28.960964	34.95069	29.79642	27.184637
33.933563	30.562105	31.959997	28.10456	29.746037	27.87126	24.467815	32.505474	27.976347	31.914633	29.314035	29.212568	28.753288	31.325104	31.075962	35.677242	32.920815	26.71727
31.123123	34.301476	30.484865	28.75259	30.59992	27.653131	30.864925	32.577583	28.964186	30.868818	28.936367	28.976246	29.194855	31.922882	30.739635	35.458717	34.30816	27.613857
34.959446	36.96967	29.638756	32.356148	35.950985	30.950891	32.948296	34.3926	32.955	31.03579	32.94247	27.4509	31.844727	29.924377	33.473114	Undetermined	33.250088	25.813427
31.481775	29.037432	28.292677	31.295317	32.31136	27.49124	29.970974	30.01508	29.510748	29.893644	30.334612	28.439318	26.970928	27.270056	29.966908	34.946587	28.710077	25.973543
Undetermined	35.242058	33.696632	33.903854	34.344456	31.160105	33.345455	34.70241	31.931425	32.38328	32.932373	30.764988	31.594112	32.476322	34.97932	Undetermined	35.946426	29.962315
32.29876	33.110367	28.43332	29.973152	31.928885	26.943972	28.942867	31.969902	27.87612	28.869524	26.892328	28.070364	28.945343	30.588999	29.951065	34.130005	32.965847	26.967766
29.686445	33.957306	29.19754	27.201105	30.305435	27.664476	25.522482	29.167803	28.59723	30.972357	29.373463	24.629358	25.958883	27.457619	28.879189	36.941963	31.072042	24.910257
28.971382	30.422794	27.697798	27.964525	29.318186	25.812407	21.766775	28.676706	26.677145	28.80608	26.848782	27.424238	25.661535	27.7866	27.929974	32.471394	30.083689	24.98344
33.95352	33.728622	32.965046	32.826584	33.95513	28.771812	27.953691	34.932392	29.954527	31.582079	30.880356	31.9798	31.070557	32.93496	32.938213	34.071045	35.996983	30.910128
27.753265	31.953772	29.955534	27.765207	29.18683	26.690128	28.95784	29.971008	26.909313	25.919954	28.350574	27.261103	26.715004	28.950169	27.29864	32.970596	30.51686	24.98529
29.970146	31.820913	26.580153	28.590237	28.944965	25.944794	26.23122	29.548094	24.934143	27.975094	26.68112	27.946566	26.950691	28.807175	28.281933	32.81199	28.800367	24.790308
30.205048	32.53196	29.172623	27.407478	29.927683	26.646517	28.649334	28.511646	26.326431	28.50426	27.913212	27.630348	26.23565	28.511602	28.339005	34.273125	29.966515	24.419758
29.568565	31.16685	26.667631	28.44827	27.602055	25.95602	27.945381	29.979351	25.962814	29.936926	27.93769	27.596151	26.534138	27.386433	28.49006	27.836575	28.279299	25.996672
29.441591	33.95498	31.465904	27.957636	30.58175	27.43843	28.420557	29.983301	27.123543	30.990488	30.53944	26.542383	26.95273	28.582748	27.775473	29.465475	30.370419	24.821001
31.076696	31.922546	29.478104	27.82694	29.498047	27.942327	22.767	29.919285	28.014957	30.991798	28.942657	26.00353	28.08813	29.506779	28.816027	31.251064	29.323484	26.645285
34.75275	37.00322	31.970535	30.263813	33.62837	29.429842	32.5361	33.02984	31.589504	31.98603	31.45752	27.702717	28.477781	31.014475	32.962658	37.066113	32.23288	26.749168
36.755295	32.269527	30.71137	31.981165	33.625076	30.739973	31.876947	33.98874	31.707596	33.332348	32.749466	28.147064	29.943132	30.928751	35.36144	34.817646	32.445137	26.727303
29.972342	27.932833	28.919298	26.56318	28.9681	26.454475	23.53783	28.97782	25.88265	30.978786	27.762753	21.468027	25.398071	27.963211	27.933453	30.986977	29.742031	23.943117
34.667408	Undetermined	33.177612	34.13938	36.93569	31.28166	34.33249	32.33211	31.377691	34.48065	36.95206	30.974274	28.98653	30.93428	33.29872	Undetermined	36.962643	25.708363
31.228704	31.100422	28.573473	29.659227	31.927141	26.859114	29.9702	31.146927	27.292883	28.98066	28.675907	29.659302	27.232927	29.544184	30.457542	34.65946	31.870186	28.12577
34.957954	34.75016	31.710928	32.70232	33.84253	29.91814	34.843735	35.963318	30.935875	33.880016	30.360262	30.96755	31.304945	33.189823	33.843456	Undetermined	Undetermined	28.839418
31.497656	34.949184	31.65113	30.959679	35.92487	29.95171	30.958649	31.033575	30.566423	32.950462	34.236294	25.212233	27.543198	29.677624	29.969831	34.84024	34.965916	26.021477
35.255802	Undetermined	33.980865	32.8994	32.9418	30.948519	33.54911	34.50866	31.566402	29.397764	32.951996	32.92006	31.586481	37.018883	34.29658	Undetermined	Undetermined	31.499578
31.098656	31.622602	28.427586	25.967773	28.245493	27.167488	23.462252	30.562578	26.489422	26.687534	27.05449	27.388596	26.958843	30.444296	27.962503	34.80029	29.969143	25.898516
33.130444	34.49255	29.969734	29.752554	33.18426	28.94166	26.945814	32.73203	29.95817	32.474407	30.968534	29.791256	29.327791	31.667341	30.720379	35.962288	33.909832	27.81923

GCLC	GCLM	GST pi	GTF2H2	hENT1	hENT2	HER2	HER3	HER4	HIF1A	HPRT	HSP60	HSP70	HSP90	IAP2	IGF1	IGF1R	IGF2
**NM_001498**	**NM_002061**	**NM_000852**	**NM_001515**	**NM_004955**	**NM_001532**	**NM_004448**	**NM_001982**	**NM_005235**	**NM_001530**	**NM_000194**	**NM_002156**	**NM_002154**	**NM_005348**	**NM_001166**	**NM_000618**	**NM_000875**	**NM_000612**
30.65278	35.98646	28.38391	31.578083	29.967266	35.978264	34.158955	28.634317	31.44381	27.930712	35.946007	32.337444	29.548967	31.413054	33.954132	29.943626	30.504484	36.296734
29.906282	31.67901	24.90705	27.914726	28.139688	32.97846	34.9903	28.95431	30.31729	25.911833	31.060602	28.882107	27.681574	25.83755	29.228182	30.665405	28.541176	32.759613
32.48911	32.463326	29.324455	32.31647	32.955746	Undetermined	32.268982	27.969772	33.438564	26.043585	34.242424	30.91249	29.202486	30.337397	33.930573	27.614674	28.832552	34.63605
23.938805	26.372362	22.963535	27.831276	27.050457	31.990913	30.171732	27.67772	32.992424	24.756227	28.803034	27.077557	25.918985	23.935549	28.87933	30.872108	26.330812	32.95996
34.97982	35.983887	28.833897	29.62961	29.422014	33.880802	34.970753	29.974123	36.463	28.964966	34.9465	31.351562	29.52112	30.561771	31.876898	31.948795	30.759136	Undetermined
26.445423	28.249994	22.438723	27.064556	26.398092	31.265516	28.968182	24.319508	32.61232	25.069527	30.577736	26.129772	26.861649	25.529793	28.411825	28.910534	25.403843	35.84899
28.92379	33.707077	24.907862	26.974167	30.229496	34.987816	35.503094	27.961363	35.294193	26.166851	30.963495	28.820574	28.355377	25.943256	30.93525	32.66371	27.924461	29.234266
32.293476	32.95485	25.652733	30.218777	29.951986	34.616898	31.861275	26.755957	34.940674	26.9315	32.84595	28.933283	28.926636	27.745905	31.459557	30.08899	28.369495	Undetermined
29.596394	31.466206	24.158998	28.970251	28.58037	32.65801	28.321404	25.669819	33.94455	25.392384	31.824535	29.126656	28.831436	27.797186	29.927185	29.506691	27.824463	30.315731
30.954597	32.612637	23.957521	30.615938	31.885504	33.299885	33.97348	28.636852	34.95293	26.342354	34.62154	30.949879	29.915983	29.185658	31.868217	29.93442	27.920769	33.13101
29.896727	31.367006	23.309296	29.702187	29.664728	34.17204	30.596016	26.931904	32.77737	25.394743	32.940117	29.425879	29.318668	29.92395	31.199205	28.684015	27.818394	32.525566
31.966368	31.732262	28.955254	32.217533	33.976578	36.98076	Undetermined	30.890917	36.926434	27.542263	Undetermined	30.773323	29.123165	30.954885	35.360565	28.503654	29.675978	36.987164
29.13179	30.975222	23.686083	27.969929	26.978167	33.971832	29.829737	25.957035	31.321253	23.935347	30.659754	28.271862	26.857943	25.284588	28.555592	29.765263	27.229568	35.970566
35.9338	Undetermined	29.707008	32.586926	32.579613	Undetermined	Undetermined	30.222534	Undetermined	27.931898	Undetermined	32.34668	30.911762	30.96048	33.60135	31.911295	31.933468	Undetermined
33.177933	35.98559	28.88635	31.973902	30.962734	Undetermined	35.95664	33.80074	34.18444	29.41745	34.95991	31.856709	30.318836	28.633354	32.91806	30.183487	30.739468	35.418472
35.940914	34.535248	26.724567	34.59681	34.646812	33.979767	33.102467	28.062527	36.949764	31.527012	Undetermined	27.938301	35.27278	30.488647	Undetermined	32.836014	31.560406	35.972958
28.168211	30.867401	24.970154	28.474686	28.928692	32.841427	30.535994	27.95167	30.983921	23.844221	30.912764	28.442282	27.574528	27.003468	29.25261	28.613596	26.79931	32.77282
27.791265	27.961922	24.728619	28.903048	29.951584	33.53329	32.039593	27.958002	36.933067	26.147345	29.830755	28.530598	27.930857	25.869856	30.466475	30.590899	27.939922	35.95369
32.968544	32.34616	25.944508	28.56284	31.791927	35.945698	33.1569	29.418669	33.575115	25.913866	32.936333	29.567913	28.547153	27.236609	30.91911	29.933096	27.93493	34.84635
31.678755	32.19391	27.933306	32.995632	32.80651	36.043285	32.136597	27.167976	33.203682	27.930864	35.29607	28.63404	30.055916	31.381636	34.943893	29.813961	28.924135	30.958965
30.189775	30.700808	27.619474	28.589409	29.46851	31.623089	31.284536	26.62826	28.721272	26.921434	32.19802	27.939043	27.921837	28.98983	32.18572	28.990425	26.93504	24.96354
34.651978	35.98229	27.963139	33.132965	32.053806	Undetermined	33.96383	28.995646	35.752064	28.406843	Undetermined	32.767982	31.17338	30.627903	35.53226	29.933811	31.17809	Undetermined
32.859108	30.864275	25.773619	27.965864	29.755007	34.317825	31.962687	26.842098	33.180252	25.166395	30.856827	28.426384	27.935383	25.959234	29.929636	27.929913	27.934631	33.544136
28.924578	27.735958	22.966862	27.93209	29.150066	29.077553	27.843426	25.78637	31.848598	26.333153	29.908348	27.94524	28.879967	27.900032	30.974195	30.297585	26.456976	32.198833
28.76205	25.951508	22.968441	27.002531	27.09136	33.60546	29.238111	27.440664	32.59203	24.45622	28.575138	28.485834	26.939365	26.40051	28.826086	28.412367	26.935095	29.659971
33.16032	33.992447	25.973518	29.955612	30.949272	Undetermined	Undetermined	27.961025	33.942207	28.929457	34.300167	32.576576	28.969656	29.96401	33.588333	32.93578	28.69752	32.984455
27.938446	28.430006	21.609238	27.956917	28.145847	30.631483	29.530666	25.329786	33.778717	24.236948	28.791634	27.663364	26.323467	25.728931	28.222841	30.358536	25.72188	29.396387
27.816717	27.718975	23.105272	25.917103	25.943892	30.637274	28.441269	25.404055	30.949295	22.92771	26.66513	24.939342	25.35831	22.746151	27.618279	26.916666	25.814512	30.970602
27.069288	29.001596	22.877308	27.840858	28.582401	29.98568	29.979658	25.96327	31.747707	24.33833	27.962221	26.695623	26.920216	24.94213	28.93768	28.386847	25.897078	29.968754
29.80108	29.643152	23.117441	27.060896	27.367418	30.893887	28.06516	24.536268	33.141884	25.403679	29.658207	27.733667	24.945679	24.69339	28.936472	29.631063	26.93404	30.257881
28.93812	30.815207	22.597988	29.720692	29.77376	31.976152	29.968456	24.93714	33.380695	26.766365	30.947132	29.946707	28.27432	29.569893	31.467346	28.66724	26.802011	29.348366
26.947327	27.022831	23.147392	28.673098	29.244349	30.56861	29.52818	25.619799	33.6433	25.708158	29.319994	28.97926	26.685423	26.96094	30.391855	30.39986	26.64884	30.31417
31.946426	32.40236	26.090956	32.04963	31.110226	33.45364	35.664803	30.214483	Undetermined	26.9209	35.65866	28.64299	29.906916	29.91971	33.321835	30.650759	29.92158	35.941082
30.6963	32.181976	26.214025	30.956915	30.053106	36.993046	32.920143	28.936209	35.946724	27.301699	34.956333	28.8867	28.627598	27.386818	33.062172	30.957031	27.507627	29.15872
26.09751	25.664333	21.960863	26.960253	27.456535	28.970135	28.902561	26.961393	33.614872	23.91574	28.942495	25.902203	26.46917	25.886793	28.192314	29.3983	26.432844	31.8967
36.93433	35.93161	24.979313	33.356087	32.496044	31.64148	31.248539	26.669403	36.9565	32.930866	Undetermined	26.944696	35.632336	29.93245	Undetermined	32.913986	30.915571	30.70472
29.719833	31.004673	26.558638	27.176464	28.971136	34.8598	33.525776	27.288473	28.06975	25.760242	29.610888	27.18738	26.802523	23.937801	28.43624	29.210823	27.920282	34.46958
31.728577	34.97501	26.799168	30.967157	32.760273	Undetermined	Undetermined	33.149864	Undetermined	26.940771	34.28149	30.490059	29.140865	28.090641	32.77635	31.033697	33.307487	Undetermined
32.930073	33.65416	24.274393	31.588985	31.985138	31.993568	31.770494	27.631102	35.944553	29.902248	34.951668	29.924765	32.201107	33.345757	35.92969	28.812656	29.399313	32.90462
35.94915	32.070686	26.09676	30.309366	35.79809	36.53183	Undetermined	30.21293	37.218464	29.464655	33.751945	31.47128	29.94654	27.946669	32.95375	32.428604	31.9201	Undetermined
23.028101	25.019745	22.71566	27.777187	29.35451	33.950905	31.964605	30.289415	36.757656	22.923376	26.904818	23.7905	25.482233	21.251543	26.931824	27.67522	26.145102	32.034866
29.785866	28.776459	24.275423	30.784586	31.948574	32.93003	32.82178	29.973656	35.51143	27.77121	34.10049	29.451048	29.918531	30.14996	32.721043	29.176641	29.918968	35.98897

IGF2R	IGFBP1	IGFBP2	Ki67	MCJ	Mcl-1	MDR1	MGMT	MLH1	MRP1	MRP2	MRP3	MRP4	MRP5	MRP6	MRP8	MSH2	MSH6
**NM_000876**	**NM_000596**	**NM_000597**	**NM_002417**	**NM_013238**	**NM_021960**	**NM_000927**	**NM_002412**	**NM_000249**	**NM_004996**	**NM_000392**	**NM_003786**	**NM_005845**	**NM_005688**	**NM_001171**	**NM_000352**	**NM_000251**	**NM_000179**
31.928951	Undetermined	24.963078	Undetermined	32.93783	31.974974	36.94381	34.223854	35.515564	34.974327	Undetermined	34.976868	33.15066	32.970375	31.96662	Undetermined	35.948547	35.187252
28.994875	36.041286	25.87772	33.16957	28.946518	29.92796	33.055508	30.775042	32.42958	30.08388	34.37994	33.641293	32.203087	28.7472	29.087872	34.95834	29.916403	31.455036
29.617662	34.431225	28.337715	36.94351	33.86241	31.964712	37.021126	33.11059	32.906284	35.922466	Undetermined	35.986916	36.23146	34.97803	32.96636	Undetermined	33.31501	32.6793
28.21273	35.384377	24.796793	28.98699	25.961796	28.50779	34.146557	30.421448	29.93626	26.969824	28.671227	28.64679	28.400774	24.607924	35.94846	Undetermined	28.120619	28.340464
32.6553	Undetermined	32.96322	Undetermined	31.04636	30.792309	35.01873	33.224113	35.964436	33.949867	Undetermined	32.396812	33.711185	30.447708	31.994171	Undetermined	35.971073	34.586178
27.012897	34.362312	22.540596	30.854654	25.847824	26.96131	32.128864	28.984018	29.018982	28.825254	27.850996	27.811766	29.739216	28.41454	27.283722	33.930836	28.080463	29.89388
29.928493	35.957222	27.175978	29.681833	28.203098	30.969269	34.778313	30.52612	31.493355	31.458689	35.218037	36.97744	34.941124	29.440989	33.36093	32.288002	28.848902	30.95761
31.302174	Undetermined	28.981476	Undetermined	28.92243	31.245064	33.969784	32.770752	32.874897	31.722162	34.94303	34.50888	30.88322	30.973543	31.348793	Undetermined	31.95058	33.34517
28.769697	Undetermined	27.504595	32.870472	28.457859	27.429928	35.414993	30.28555	30.92903	29.329132	34.399864	29.213509	31.884996	28.304869	29.97817	Undetermined	30.17516	30.323505
31.318583	Undetermined	26.152075	34.91722	29.497309	30.071558	36.948536	31.888933	32.834328	30.526608	36.936436	35.96961	33.948505	29.455112	33.51761	Undetermined	31.930714	31.981173
29.934576	31.394733	28.935808	35.5729	27.957603	27.348541	35.518417	30.52123	31.67024	28.973766	32.160934	31.39899	32.867504	29.989801	31.3633	Undetermined	31.544504	30.969856
31.971952	Undetermined	29.311823	Undetermined	33.141827	34.01116	36.99786	Undetermined	35.007893	35.9849	Undetermined	Undetermined	35.946503	33.596962	34.974064	Undetermined	35.186398	33.947906
28.31111	32.958305	28.990932	31.87104	26.960203	27.951479	32.741077	29.955612	30.139904	29.965553	33.782757	28.597027	31.332224	27.914948	29.773445	Undetermined	29.477173	31.506422
33.93591	35.779907	32.407776	Undetermined	30.795298	33.274837	34.944786	36.982788	35.9153	35.965725	Undetermined	35.965855	35.4476	34.299973	33.953	Undetermined	34.297752	35.93505
33.30196	Undetermined	31.64191	35.87977	29.821115	33.963657	35.65419	32.559265	35.008087	33.25013	Undetermined	Undetermined	35.765213	32.93044	35.597458	Undetermined	33.36881	32.659912
33.850292	Undetermined	28.27511	Undetermined	35.912792	30.473095	Undetermined	35.34999	Undetermined	30.17772	Undetermined	Undetermined	35.942165	29.87305	33.84345	Undetermined	35.93727	32.761925
28.887125	35.948395	25.980293	31.473217	26.37012	27.91849	32.583126	28.798738	30.650963	28.905895	34.93891	31.756361	31.047865	27.954899	32.692028	Undetermined	28.478504	28.87464
31.456722	Undetermined	27.471798	31.922705	27.397318	31.948946	35.95753	31.835482	32.664963	28.976255	28.680462	31.678406	30.953806	28.45212	33.960854	Undetermined	28.932634	31.949333
31.944653	Undetermined	27.778261	32.952087	27.958078	32.251038	32.92641	32.977337	32.951946	30.827158	35.950542	Undetermined	32.80218	27.966816	34.885174	Undetermined	30.449205	32.349556
30.915514	34.695625	28.2807	35.955723	32.947643	32.574112	Undetermined	35.688416	33.303005	34.950333	Undetermined	35.961697	35.880787	33.021168	34.73795	Undetermined	32.85471	31.979935
28.782135	34.998173	26.810799	35.33942	30.16082	27.46672	30.689135	30.98058	30.25976	32.37382	35.959255	32.916897	33.304943	28.214188	28.87304	33.939472	31.155724	29.861525
33.122917	36.006325	32.17461	Undetermined	33.22431	33.992596	Undetermined	37.021896	34.42486	34.97828	37.13746	Undetermined	36.00141	34.83427	34.078304	Undetermined	33.865402	34.55174
31.935968	Undetermined	30.760778	34.30878	26.964725	30.864336	33.113106	32.32329	32.737247	30.337126	35.26605	31.980137	32.51941	30.266975	29.911627	Undetermined	29.944654	31.968704
28.948706	35.985626	23.980343	35.06248	27.43498	27.786253	Undetermined	29.629757	32.50705	25.339514	31.404623	29.449785	31.813751	25.408838	30.540756	Undetermined	30.05299	28.241257
27.93157	33.52721	26.693865	31.977259	26.142052	26.062572	Undetermined	29.09198	30.592989	26.981052	28.902077	28.97335	30.963161	26.972456	30.299593	32.3811	28.624134	27.960503
33.921276	Undetermined	29.32918	Undetermined	28.96847	32.940746	34.228947	33.930042	32.929302	30.95918	35.926388	34.980427	31.056946	30.960655	32.485386	Undetermined	32.563007	34.965622
28.63773	31.982805	24.502842	30.892275	26.751442	26.905876	34.2842	28.98606	30.941324	26.682693	33.948055	32.264687	29.556072	26.410683	31.948795	Undetermined	27.85418	27.109915
27.556711	34.436436	24.612787	28.43735	24.961765	26.971104	31.31748	27.345749	29.728802	27.349022	33.44814	32.52754	28.81324	27.370693	29.042637	34.942795	26.903095	27.430834
28.622974	36.012096	23.979063	30.33553	27.766441	27.049181	33.93604	28.661333	30.96297	26.706999	33.77615	34.299076	30.401155	25.546143	30.855085	Undetermined	27.956	28.642925
29.02333	32.99496	25.986368	30.653448	26.813982	26.360052	30.656977	29.232557	28.933651	28.008524	33.248158	28.132002	24.69638	27.383467	28.965014	Undetermined	28.320543	29.425928
30.009268	Undetermined	29.420055	32.930805	28.186827	24.933182	37.057293	29.843487	31.77552	26.741318	31.721573	32.09334	28.806873	25.084879	30.96246	Undetermined	30.947704	28.72306
29.364021	34.14233	28.283161	31.721508	26.96907	26.739159	Undetermined	30.953955	31.285105	26.358961	26.938572	30.044844	27.238832	24.825085	32.647457	Undetermined	29.579565	28.980356
30.235432	Undetermined	26.95942	34.681835	30.948246	30.493454	33.96301	32.355885	34.269936	32.272655	Undetermined	Undetermined	34.838585	31.087934	32.14142	Undetermined	34.922653	32.799652
30.922739	34.516117	28.071882	32.729984	31.603054	32.682617	35.026012	32.117702	32.250443	35.63331	Undetermined	34.97692	35.940643	34.963654	35.941814	Undetermined	31.248602	31.975445
27.9377	35.984974	24.013578	30.2777	26.607433	26.486464	34.613953	28.775957	29.945425	26.518297	28.185339	29.972012	29.954361	26.478981	32.812607	Undetermined	24.654621	26.006104
35.395683	35.119152	28.99852	Undetermined	32.367443	30.973436	Undetermined	35.314545	Undetermined	31.542446	Undetermined	Undetermined	36.80525	33.015347	33.132843	35.945076	37.162655	31.828133
29.956137	35.013546	28.56794	33.511993	27.326271	29.95125	29.97622	30.444632	29.989513	31.5025	33.690273	27.953651	30.479542	28.736582	32.819263	30.876568	28.94769	30.636145
33.993206	Undetermined	33.969707	Undetermined	31.195486	34.191467	Undetermined	35.94749	34.46179	33.96953	35.72553	30.76676	35.971336	32.97004	35.97767	Undetermined	33.480885	35.2438
31.889206	Undetermined	29.179499	Undetermined	30.56429	27.978909	Undetermined	32.696568	36.929043	29.188353	35.95273	37.128605	34.61085	28.948172	32.71849	Undetermined	34.55931	31.190191
36.96329	35.249763	33.412724	Undetermined	29.177147	Undetermined	Undetermined	34.91698	35.141823	33.2961	36.96591	Undetermined	34.947273	33.129177	Undetermined	Undetermined	32.677273	Undetermined
28.93509	34.68949	24.476505	28.924475	26.662743	27.716047	31.80656	30.395359	29.976416	26.96649	30.068565	30.005722	29.227652	27.541216	33.955063	Undetermined	24.715246	28.279587
31.436304	Undetermined	28.480928	36.569473	29.954893	30.678722	36.94384	33.9473	33.667088	29.630238	29.947716	32.98306	33.10519	28.334332	33.254417	Undetermined	29.87072	31.956053

MTII	mTOR	MVP	NFkB	OPRT	p16	p21	p27	p53	PBGD	PIK3CA	PTEN	Rad51	RRM1	SDHA	SOD1	STAT3	Survivin
**NM_005953**	**NM_004958**	**NM_005115**	**NM_003998**	**NM_000373**	**NM_000077**	**NM_000389**	**NM_004064**	**NM_000546**	**NM_000190**	**NM_006218**	**NM_000314**	**NM_133487**	**NM_001033**	**NM_004168**	**NM_000454**	**NM_139276**	**NM_001168**
29.962408	33.321762	27.97558	30.971024	Undetermined	32.436333	28.974901	28.022068	32.19223	31.644325	33.466095	Undetermined	34.112137	33.571045	32.078617	27.832817	28.965492	34.943974
24.664858	31.227728	26.486126	28.19563	33.27642	28.399717	27.912933	26.724525	30.95959	29.959152	30.80683	Undetermined	29.994587	28.139872	30.463211	26.903961	27.969719	29.98669
31.00302	31.056969	28.937626	32.95915	34.26061	30.47266	27.952621	26.776405	31.002958	30.924198	33.720932	36.01254	32.19797	32.060818	29.944525	26.761955	27.495974	36.93921
25.942295	29.736126	26.392607	28.266281	31.52632	33.64612	26.712067	25.633284	28.1746	28.485266	28.20628	30.915688	27.637745	27.19787	26.638718	23.78827	26.805656	29.57397
26.822767	33.974342	26.218904	31.014791	Undetermined	34.981976	30.004053	28.825905	33.98614	32.96261	35.9598	37.01496	35.8346	32.938915	33.945377	27.962965	29.984476	33.89724
24.99381	28.682379	23.419338	26.024231	31.839396	30.72843	27.603762	25.965143	28.458267	28.217262	30.979393	32.123928	28.94901	27.935953	26.625826	24.259172	25.405762	32.34326
25.644823	30.741879	28.41992	29.94791	33.929806	27.951046	27.9744	25.827135	28.966825	29.945957	32.90557	36.03493	29.197636	27.470947	29.443714	26.61928	27.959797	30.9705
25.400793	31.927105	26.957544	28.98603	35.456898	33.35883	29.66406	27.683432	31.667356	29.536608	33.519756	31.74614	31.449492	30.900175	30.929611	25.959253	27.945887	35.8198
25.531582	30.846746	24.957615	27.780516	34.930103	29.224	27.809217	26.799889	29.669764	28.936415	32.4752	33.166492	30.726458	29.45106	27.408792	26.554815	26.629248	32.130447
27.179392	32.881496	26.651426	27.96397	Undetermined	31.154541	27.957	27.75767	30.825983	29.905592	34.67626	35.96853	29.980415	29.934742	29.984589	27.658476	27.95704	32.94918
26.51154	31.542713	25.437735	27.59761	Undetermined	31.935211	27.900229	26.94296	30.953884	28.88627	33.95028	31.966805	31.979342	30.661757	28.39519	26.940685	26.403189	32.725735
28.53989	33.56506	29.964626	32.839016	36.935074	33.50354	28.46661	27.938423	32.18343	32.09959	32.967903	Undetermined	33.97527	32.0011	35.35268	27.983133	29.431768	Undetermined
25.98105	29.961508	24.340755	27.870817	32.819237	33.229267	26.28201	25.678036	29.981836	28.752165	31.933193	32.849503	30.97985	28.899502	26.89734	24.943842	26.55999	32.271065
27.54167	33.943935	29.31601	32.963543	Undetermined	34.958942	29.85496	29.951872	35.977077	33.28396	36.917557	Undetermined	35.051918	33.94596	33.981056	29.350164	31.294832	Undetermined
28.94745	32.80745	29.947556	31.72855	36.94058	Undetermined	29.756794	29.701157	34.614143	33.15529	33.17178	Undetermined	35.94044	32.41079	30.775797	28.290606	30.955608	Undetermined
28.195017	35.89595	28.503868	29.607925	Undetermined	35.64057	31.965921	32.315464	34.953262	31.925253	Undetermined	31.962208	34.956223	35.737663	33.75864	30.47843	27.807781	34.962563
25.675814	29.412077	25.385475	27.728245	31.994482	27.216867	26.809404	25.35237	29.634825	29.43223	31.035471	Undetermined	28.631805	28.574871	27.755493	25.9386	26.48479	31.299185
25.402754	30.759293	27.640419	28.769978	33.48767	29.939129	28.591398	26.937494	30.67598	29.45379	31.562119	Undetermined	28.976425	29.943182	31.605328	25.88264	28.04165	32.435043
26.958103	31.848259	27.15652	28.614403	Undetermined	35.37849	28.81404	27.331257	30.943193	29.937548	31.733639	Undetermined	30.470919	30.370115	32.558884	27.455883	27.956541	32.9035
29.96343	32.33908	28.25061	31.962322	Undetermined	30.519606	27.521389	27.700619	30.126211	30.581312	33.58125	31.816559	32.242847	30.100027	31.3478	27.606544	27.492556	35.952065
28.21167	30.502499	25.487871	28.640488	Undetermined	31.91603	26.422247	25.35119	29.630735	30.96753	31.98256	29.118298	32.930122	29.459745	27.66696	25.629015	25.984137	35.718544
32.57602	32.52896	28.963953	32.558712	37.15324	37.0124	29.57698	28.925663	33.255024	32.87688	34.25987	Undetermined	32.874493	33.243412	31.752155	29.58974	29.240677	Undetermined
25.26011	30.156006	24.947598	27.669565	35.148823	32.407043	27.96476	26.875504	31.082159	28.818502	31.94894	35.943058	30.968184	29.909473	30.948244	26.622124	27.700443	34.977764
26.943855	30.944681	24.812134	26.955343	36.965553	28.211044	28.602219	26.926125	29.710644	27.245407	31.949615	28.5318	28.668987	27.893938	28.188253	25.961193	25.72878	30.984085
25.04298	29.63013	23.18371	25.03381	34.81293	34.44591	26.819618	26.258743	27.592428	26.837772	31.456295	29.831003	28.946821	27.939142	27.757202	24.52177	25.011524	30.159323
28.942913	33.96878	28.608477	29.762491	Undetermined	35.94441	29.6305	28.940466	31.973557	29.938688	34.331375	Undetermined	33.377125	32.112034	33.0278	27.97138	29.762598	Undetermined
24.80015	29.511227	24.515257	25.946987	33.41011	27.95261	25.613949	25.8014	29.978193	28.144026	29.50912	31.433693	27.754572	26.602196	26.924934	24.699799	24.980726	29.954996
23.31567	28.040524	23.955418	26.39105	32.946373	32.50835	25.969353	24.943151	28.85778	25.939856	28.572477	33.01371	27.55306	27.412828	26.977348	23.949492	25.003706	29.455587
23.960451	28.943518	24.970036	26.244864	33.566364	27.760885	25.974878	25.82796	27.983746	27.51418	28.945805	32.551125	27.459095	26.748539	27.83119	24.806139	26.048979	28.458263
23.93257	29.389158	23.947641	25.982954	33.858192	29.90098	26.693367	25.785057	28.462103	27.246264	30.519957	36.99102	28.953327	28.448715	27.962818	25.866106	25.988478	31.52397
28.859941	33.486786	24.351576	26.530684	36.92475	29.650726	27.797588	26.968758	28.732046	27.467037	33.922443	31.072437	28.98091	28.939157	29.508276	27.547457	26.303093	31.979757
26.066063	31.530733	25.164564	26.954435	34.531647	32.650112	27.96328	26.78326	28.819462	29.710987	30.905294	33.992752	29.354187	28.852169	27.555372	25.956415	26.61359	31.719774
28.899693	31.870302	26.906437	31.148493	Undetermined	27.962587	28.195755	27.756958	32.97357	30.932934	35.309803	32.979664	31.955978	31.16631	29.901814	27.9439	28.469843	33.2074
28.954662	31.940165	28.973818	32.74365	34.352856	27.70387	28.983725	26.784843	33.658573	30.892445	33.95825	Undetermined	31.35476	30.935041	30.063837	26.469072	28.949114	30.9285
24.955505	28.049597	24.640667	26.942554	32.397392	31.94456	26.731216	25.933737	27.77134	27.344494	29.945473	31.677574	27.421255	26.937172	25.681528	24.799177	24.945473	28.954561
28.526417	36.881546	26.412296	28.647526	Undetermined	Undetermined	31.659136	33.553246	31.650297	30.793135	Undetermined	30.983812	35.93082	34.69304	36.96661	31.833233	26.383108	Undetermined
27.619905	29.955347	26.761017	28.076708	33.520485	34.944866	27.454462	25.575405	30.955378	30.751625	30.250128	36.002064	31.703213	29.522373	29.513552	25.943964	26.998087	33.970116
28.21784	35.13888	28.912033	31.11295	Undetermined	Undetermined	30.159155	29.962814	33.852146	31.787128	34.930298	Undetermined	33.612255	33.6301	33.912384	28.435095	30.378225	35.718987
30.82013	35.152546	25.764555	28.04048	Undetermined	33.362507	28.98909	29.934113	31.329845	29.92548	35.90685	31.335947	32.571255	32.773903	32.811275	29.7901	26.834656	34.962498
28.970785	34.881393	32.39353	31.479729	Undetermined	Undetermined	32.99331	31.93892	33.985985	33.181877	37.004257	Undetermined	32.07525	32.851357	Undetermined	29.962206	31.880974	34.244343
22.816225	28.514206	26.627449	26.812523	30.926168	26.390476	25.963583	25.655384	30.520285	28.479406	28.459953	34.862392	27.505077	26.469767	26.670025	24.850466	26.360437	28.900717
29.078693	32.08874	26.952868	29.709215	Undetermined	30.891533	29.349812	28.873493	31.973448	30.927082	33.74797	33.93798	31.171516	31.834389	30.162659	27.967876	28.231764	33.95845

TAP1	TAP2	TAP4	TBP	Topo I	Topo IIa	Topo IIb	TS	VEGF	XIAP	XPA	XRCC1	XRCC5	XRCC6
**NM_000593**	**NM_000544**	**NM_000443**	**NM_003194**	**NM_003286**	**NM_001067**	**NM_001068**	**NM_001071**	**NM_001025366**	**NM_001167**	**NM_000380**	**NM_006297**	**NM_021141**	**NM_001469**
31.989246	31.708282	Undetermined	34.449886	32.23805	36.927822	29.53466	34.51271	30.526436	34.215023	31.723518	32.8155	28.98843	26.99527
27.020311	28.48929	32.52358	30.693243	28.896301	28.081104	26.694838	29.38104	28.966843	29.995153	30.254486	29.073896	27.795568	25.967005
31.994171	32.614098	Undetermined	32.841755	33.477097	36.95489	31.903547	34.897335	28.121754	34.460693	29.0868	35.64084	27.957245	25.98628
27.620062	28.501434	36.99367	29.640278	27.898216	27.934883	25.979172	28.911234	26.959887	28.923525	27.964767	27.33152	25.963552	24.453907
29.394665	30.554958	Undetermined	33.61538	30.384083	30.339396	28.338358	35.2798	31.98613	32.027206	32.999683	34.631382	29.980776	27.976376
26.946352	27.870369	31.384697	29.730217	26.444498	29.486536	25.979746	30.159155	27.442368	28.697811	28.168848	27.041151	25.111134	24.417665
27.569435	29.6964	32.94737	29.917988	28.837582	27.677073	26.950815	28.357712	27.799454	30.9271	30.225016	32.09198	25.9598	25.967104
29.76389	30.973692	34.307842	31.539742	30.939095	34.68298	27.935444	33.624363	29.110718	31.889622	31.621492	29.355091	28.035282	27.354404
27.390862	28.794031	34.942028	31.71358	27.92089	31.07477	27.716406	31.935497	26.543215	29.941835	29.8715	31.00407	25.950277	25.711967
27.659182	28.929342	36.930866	32.686295	30.905193	32.631386	28.502117	32.68422	28.616886	32.262054	30.974766	30.435518	26.972353	26.69503
27.247072	28.124702	35.685673	31.941513	29.876904	32.793007	28.679012	32.577896	27.976215	31.067322	30.48931	28.493864	26.385485	26.439928
32.26875	32.961628	36.988068	33.49778	34.928738	36.899532	30.780052	36.379192	30.61465	34.729004	32.53489	Undetermined	28.744238	27.872543
26.962666	27.960672	33.765224	29.970161	27.825619	30.933496	25.93251	31.540812	27.90151	28.938486	28.625397	28.733738	25.671375	24.95917
31.69976	33.962803	Undetermined	35.970127	31.919281	33.436214	29.929136	35.754833	30.948902	34.972775	32.738094	35.64598	30.74531	28.560213
31.968021	32.666035	Undetermined	34.569355	32.84291	37.12513	30.182596	Undetermined	31.608507	34.41442	31.442673	35.477962	30.492435	28.841818
32.392067	31.80275	Undetermined	Undetermined	34.7319	Undetermined	33.509678	36.93636	29.915035	Undetermined	35.978027	32.669918	29.96345	28.971514
26.855806	27.963236	33.960182	30.948305	27.933825	27.301884	26.647217	30.266665	26.401487	29.940714	28.1072	30.974136	26.66843	24.827063
28.961786	30.253284	36.976494	31.39687	29.513021	30.268023	26.67883	30.315805	29.407991	30.9213	32.599648	31.960014	27.874783	25.367382
28.722286	29.896841	33.78924	31.930454	30.88647	31.945505	27.100758	32.66763	30.629852	31.869736	32.97633	31.694742	27.336592	25.963503
30.97025	31.960043	Undetermined	32.19367	33.61302	33.493855	31.950888	35.92027	28.18433	Undetermined	30.775196	33.924786	26.81771	26.443497
27.112385	27.845482	32.648193	29.938316	30.899248	30.832003	27.738232	32.328896	27.075796	31.948803	26.946287	28.261673	25.68113	25.089302
32.761375	33.81368	Undetermined	34.338062	34.578114	Undetermined	31.9463	36.91999	31.020388	34.86631	33.69089	Undetermined	29.183044	28.0448
27.851828	28.97145	Undetermined	31.88421	28.86831	31.582287	26.93351	32.641983	27.9521	30.39199	31.571814	30.982527	26.969152	25.925932
28.37022	28.129333	34.10121	30.759312	29.039522	30.90541	28.013245	31.918406	26.750475	31.981987	30.024721	28.686665	25.885849	24.94906
25.97105	26.092588	32.734974	30.683	27.899435	29.506716	26.711058	29.766218	25.584309	30.313267	28.733177	25.973778	25.66706	25.025637
29.929857	30.874971	36.953373	33.927505	31.88593	28.421368	28.146147	Undetermined	28.957941	35.7356	31.921503	Undetermined	29.966175	27.948112
25.722597	26.598001	35.38339	29.371677	27.721048	28.161701	26.931723	27.93158	24.967104	29.963879	29.895979	28.959955	25.924063	24.963179
26.381823	26.97091	33.37103	28.723288	26.79198	26.927732	25.257631	28.376795	24.966763	27.9176	28.976957	26.872307	24.670034	24.070204
26.983969	27.403034	33.953728	29.940647	27.721088	28.335304	26.025133	29.495981	25.522806	29.93872	29.229927	27.3874	25.964174	24.489508
25.972303	26.956276	32.525997	29.654167	26.907473	26.221678	26.488815	30.451317	25.849588	29.180346	27.13311	26.848541	25.978271	24.226374
27.961206	27.413446	35.429905	31.855959	30.580404	Undetermined	28.935606	33.608845	24.999298	31.8804	28.016817	27.352455	26.634504	26.072826
27.349806	27.945496	34.869896	30.747776	29.490908	27.204195	27.921421	31.959244	26.231554	30.935575	27.87728	30.040262	27.416704	25.973648
30.66463	30.720015	Undetermined	32.945072	31.928835	32.128395	30.366568	33.242393	29.968485	32.957203	31.988653	33.37176	28.758392	26.945557
31.938076	32.973907	Undetermined	32.723053	31.914948	31.215363	29.647562	33.10374	29.923777	33.264908	31.649982	34.97941	28.600042	26.157867
25.9702	27.523062	33.202923	29.176727	27.32739	27.949512	26.280857	29.949678	27.326355	28.60224	28.40853	29.233784	25.525711	23.75166
31.971409	32.90211	36.96008	36.942337	35.910583	Undetermined	34.92617	Undetermined	28.46313	Undetermined	34.91871	32.1492	28.978323	27.952915
28.436346	30.981913	34.532795	30.40505	28.886616	28.528519	25.942688	32.987278	28.96254	28.94563	28.698112	29.151043	26.60305	24.9691
30.138563	31.834768	36.936035	34.65428	33.19502	31.833366	29.882286	32.1631	32.782883	34.38128	32.95883	33.88702	29.6837	28.118425
29.966555	29.741652	35.762554	33.93405	34.486507	35.96947	32.59688	34.9258	28.561327	34.99481	32.68429	31.21049	27.952435	28.32828
31.515175	35.094624	Undetermined	34.943306	34.107693	34.41137	30.469694	33.842773	31.048735	35.743286	34.649384	35.967056	30.269209	28.874025
26.92365	29.97089	31.955585	29.424538	27.539015	26.81747	25.61987	28.75917	25.95683	26.877556	28.710335	30.95702	25.94323	23.348915
29.589556	30.655993	Undetermined	32.303524	31.686613	33.951862	30.3764	34.62987	28.851149	33.9779	31.973772	33.249004	28.858095	26.928545
